# Scaffolds from Self-Assembling Tetrapeptides Support 3D Spreading, Osteogenic Differentiation, and Angiogenesis of Mesenchymal Stem Cells

**DOI:** 10.1021/acs.biomac.1c00205

**Published:** 2021-04-28

**Authors:** Salwa Alshehri, Hepi H. Susapto, Charlotte A. E. Hauser

**Affiliations:** ^†^Laboratory for Nanomedicine, Division of Biological and Environmental Science and Engineering and ^‡^Computational Bioscience Research Center (CBRC), King Abdullah University of Science and Technology, Thuwal 23955-6900, Kingdom of Saudi Arabia

## Abstract

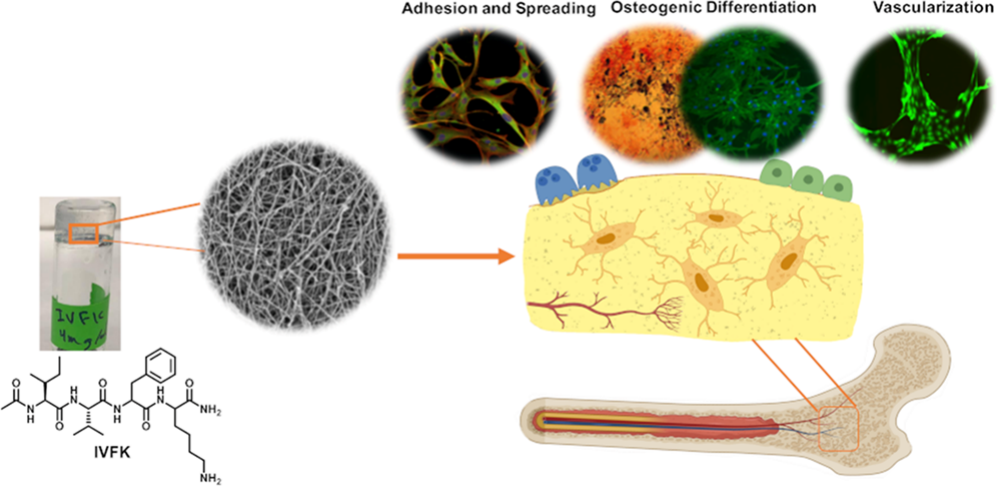

The apparent rise of bone disorders demands advanced treatment protocols involving tissue engineering. Here, we describe self-assembling tetrapeptide scaffolds for the growth and osteogenic differentiation of human mesenchymal stem cells (hMSCs). The rationally designed peptides are synthetic amphiphilic self-assembling peptides composed of four amino acids that are nontoxic. These tetrapeptides can quickly solidify to nanofibrous hydrogels that resemble the extracellular matrix and provide a three-dimensional (3D) environment for cells with suitable mechanical properties. Furthermore, we can easily tune the stiffness of these peptide hydrogels by just increasing the peptide concentration, thus providing a wide range of peptide hydrogels with different stiffnesses for 3D cell culture applications. Since successful bone regeneration requires both osteogenesis and vascularization, our scaffold was found to be able to promote angiogenesis of human umbilical vein endothelial cells (HUVECs) *in vitro*. The results presented suggest that ultrashort peptide hydrogels are promising candidates for applications in bone tissue engineering.

## Introduction

Bone is a solid organ that undergoes calcification and forms the body’s skeletal tissue. Considerable loss of bone density occurs as a result of trauma, disease, infection, and aging. Therefore, bone correction is often needed. This process is mostly done through surgery, implementing metal or ceramic implants and grafts.^1^ More than 1.5 million bone grafts are fabricated annually.^2^ There are three primary types of bone grafts used, *i.e*., autografts, allografts, and xenografts, with all having serious drawbacks. These include running the risk of donor-site morbidity, infection, blood loss, immune rejection, pain, different rates of resorption, and poor performance in some clinical procedures.^3−6^ To overcome these challenges, tissue defects have been treated through tissue engineering strategies.^7^ The subject of bone tissue engineering includes the use of cells, biomaterials, and suitable growth factors to create an ideal environment that promotes bone tissue growth and regeneration.^8,9^

Bone-marrow-derived mesenchymal stem cells (BM-MSCs) have emerged as key players in tissue engineering and regenerative medicine because of their multipotency. They have the ability to readily produce progenitors for many cell types, such as osteocytes, chondrocytes, adipocytes, and myocytes.^10^ In addition to this, BM-MSCs avoid the ethical questions that arise from the use of embryonic stem cells, are reported to have immune-suppressive effects, and are easy to isolate, culture, and expand.^11,12^ In the context of bone tissue engineering, BM-MSCs have been reported to have the capability to differentiate into the osteogenic lineage *in vitro* if cultured with media supplemented with appropriate differentiation cocktail.^13^

Scaffolds have played an important role in the repair and regeneration of a wide range of tissue types. These structures provide a supportive matrix and an essential environment for cells to spread, migrate, grow, and differentiate into specific lineages.^12^ Naturally derived materials like tumor-derived basement membrane matrix gel (Matrigel),^14−16^ collagen,^17,18^ can enhance cell and tissue function and regeneration. Nonetheless, there are questions about the safety of Matrigel in the possibility to use it for clinical purposes because its components are originated from Engelbreth–Holm–Swarm sarcomas^19^ and because it is established that Matrigel and the basement membrane matrix promote tumor growth and tumorigenesis *in vivo*.^20,21^ On the other hand, collagen, an essential component found in the extracellular matrix (ECM), has been extensively utilized as a supportive compound in tissue engineering scaffolds because it shows no or low inflammatory responses, low antigenicity, and biodegradability.^22^ However, collagen matrices lack sufficient mechanical strength and degrade quite quickly.^23^

Bone scaffolds should have the necessary osteoinductive and osteoconductive properties and good mechanical strength to direct neighboring cells to ectopic bone formation in the area of interest.^24−26^ Several scaffolds have been tested to temporarily fill bone defects, which need an additional surgery for replacement or removal. The design of a nanofibrous scaffold, capable of guiding the osteogenic differentiation of BM-MSCs, is a promising strategy to achieve clinically successful bone grafts.^12,27^ As autogenous peptides exist naturally within the human body, their nontoxic and biocompatible nature should come as no surprise. Peptides have all of the molecular information required to form well-ordered nanostructures.^28^ These materials can be designed to have bioactive epitopes to enhance adhesion,^29^ migration,^30^ and differentiation^31^ and other biological functions such as mineralization.^32^ However, recreating the ECM’s complexity, diversity, and dynamic existence remains an unresolved issue.^33^ Also, because of their low mechanical properties, the use of hydrogels in bone tissue engineering is limited.^34^ Nowadays, self-assembling peptides have gained attention in regenerative medicine including bone regeneration. Recently, the HA-Tyr/RGDS-PA/osteo-PA/angio-PA hydrogel was found to successfully promote human adipose-derived mesenchymal stem cell (h-AMSC) adhesion and osteoblastic differentiation and support human umbilical vein endothelial cells (HUVECs) to grow into vascular tubules.^33^ Another group reported that the E1Y9 (Ac-E-YEYKYEYKY-NH2) amphiphilic peptide can be self-assembled into fibers in the presence of the Ca^2+^ ion. These peptides are found to stimulate osteoblast cell growth as well as differentiation.^35^ Furthermore, RATEA16 loading with the vascular endothelial growth factor (VEGF) and bone morphogenetic protein 2 (BMP-2) was reported to support cell proliferation, migration, and tube formation of HUVECs as well as osteogenesis of human apical papilla stem cells (SCAPs).^36^ However, all of these hydrogels were functionalized with bioactive sequences to enhance their ability to stimulate osteogenic differentiation.

RADA16 is one of the most widely used self-assembling peptides for three-dimensional (3D) cell cultures. It was successfully investigated to achieve new bone formation and support osteogenic differentiation.^37−40^ Due to its acidity, the pH of the self-assembled RADA16 hydrogel needs to be equilibrated to physiological pH prior to cell seeding or *in vivo* transplantation by immediately adding a large amount of media.^41−43^ In contrast, under physiological conditions and a specific concentration, our peptides can quickly solidify and provide a 3D environment that supports cell growth, migration, proliferation, and differentiation.^44,45^ These peptides need a low concentration to quickly form a gel with good mechanical properties. Furthermore, we can easily tune the stiffness of these peptide hydrogels by increasing the peptide concentration, which provides a wide range of hydrogels with different stiffnesses for 3D cell culture applications.

In this study, we used our previously developed hydrogels made from amphiphilic ultrashort peptides^44^ that self-assemble into nanofibrous scaffolds, which are excellent candidates for use in tissue engineering applications.^46,47^ The resulting hydrogels are biocompatible and quickly gel to provide a 3D structure similar to that of the extracellular matrix (ECM).^48,49^ The aim of the current study is to investigate the efficacy of ultrashort peptide hydrogels in supporting the adhesion, spread, proliferation, and osteogenic differentiation of BM-MSCs. The osteogenic differentiation of hMSCs by these hydrogels was evaluated by investigating mineralization, alkaline phosphatase (ALP) production, osteocalcin, and osteogenic gene expression of hMSCs. In addition, the effect of the mechanical stimuli of the matrix was studied by testing the efficiency of the scaffold with two different stiffnesses to support the differentiation of hMSCs toward osteocytes. Given that successful bone regeneration needs both good osteogenesis and vascularization, providing scaffolds that can support both osteogenic and angiogenic properties is much required. In this investigation, the angiogenic properties of tetrapeptide hydrogels were investigated using human umbilical vein endothelial cells (HUVECs). To the best of our knowledge, this is the first report of BM-MSC osteogenic differentiation in these ultrashort tetrapeptides for applications in bone tissue engineering.

## Experimental Section

### Peptide Synthesis

The peptide sequences Ac-Ile-Val-Phe-Lys-NH_2_ (IVFK) and Ac-Ile-Val-Cha-Lys-NH_2_ (IVZK) were synthesized using Fmoc-based solid-phase peptide synthesis (SPPS)^44^ and purified using liquid chromatography–mass spectroscopy (LC–MS). Detailed description is provided in the Experimental Section, Supporting Information.

### Hydrogel Formation and Characterization

The lyophilized peptide powders were dissolved in Milli-Q water by vortexing into a clear solution at room temperature. Then, 10× phosphate-buffered saline (PBS) was added to the aqueous peptide solution at a final volume ratio of peptide solution to 10× PBS of 9:1. The vial inversion test was performed with different peptide concentrations to find the critical gelation concentration (CGC). To study the spatial structure of the peptide solution during the assembly process, two-dimensional (2D) NMR was performed using Bruker Avance III 600 MHz. Furthermore, scanning electron microscopy (SEM) was performed to visualize the morphology of the self-assembled nanofibers. More details are described in the Experimental Section, Supporting Information.

### Mechanical Characterization of Hydrogel Stiffness

The oscillatory rheological test was performed to determine the mechanical properties of the peptide hydrogels. The peptide hydrogels were measured on a TA Ares G2 rheometer with an 8 mm parallel-plate geometry and a 1.5 mm gap distance at a temperature of 22 °C. All of the hydrogels were made inside a Sigmacote-coated glass ring with 9 mm inner diameter 19 h prior to measurement. Six replicates with a volume of 150 μL were prepared for each sample. The measurement was performed for 5 min with constant angular frequency and strain at 1 Hz and 0.1%.

### Cell Culture of Human Bone Marrow Mesenchymal Stem Cells

Cells were cultured in a medium and supplemented with mesenchymal cell growth supplements. The cells were maintained in either a T75 or a T150 cell culture flask at 37 °C in a humidified incubator with 95% air and 5% CO_2_. The cells were subcultured when cells reached approximately 80% confluency by trypsin. The culture medium was changed every 2–3 days.

### Characterization and Preparation of 3D Culture of Human-Bone-Marrow-Derived Mesenchymal Stem Cells (hBM-MSCs)

The hBM-MSCs were cultured in T75 flasks and incubated in a CO_2_ incubator maintained at 37 °C with 5% CO_2_. Culture media were replaced every 2–3 days until the cells reached 80% confluency. Confluent cells were trypsinized and subcultured, and cells at passage 3–6 were used for the study. For the 3D culture, different peptides were sterilized by exposure to UV light for 30 min. Then, 200 μL of 3D constructs in the 48-well plate was formed by mixing the peptide solution (IVZK = 3 mg/mL (5.42 mM); IVFK = 4 mg/mL (7.31 mM)) with 40 000 cells suspended in 2× PBS. Culture plates were incubated for 5 min at 37 °C, and the complete medium was added carefully to the culture plates. The constructs were then cultured with osteogenic induction media or basal stem cell growth media. The morphology, cell proliferation, and mineralization of the cells in each scaffold were analyzed and compared. The efficiency of osteogenic differentiation was also compared with a traditional 2D culture. As positive controls, cells cultured in a collagen scaffold were used because Matrigel degrades after 2 weeks due to which we cannot keep it for the entire differentiation time (3–4 weeks). Also, collagen is considered as a positive 3D scaffold in osteogenic differentiation. The negative control was the cell from the same passage grown in a basal medium without osteogenic supplements. alamarBlue and CellTiter-Glo luminescent 3D cell viability assays were performed to evaluate the cytotoxicity and proliferation of cells. Flow cytometry was performed to study the expression of stem cell markers. Detailed information is provided in the Experimental Section, Supporting Information.

### Cell Invasion Assay

A previous cell invasion assay protocol was followed.^50^ Briefly, cells (30 000) were added to 2 μL of fibrin solution (2 mg/mL fibrinogen and 2.5 U/mL thrombin). The clusters were incubated for 30 min at 37 °C for polymerization. Then, clusters were transferred into a peptide gel (20 μL) by placing them inside the gel. The gel was made by mixing 10 μL of peptide solution and 10 μL of PBS 2× and incubated for 15 min for solidification. Cells were imaged to quantify cell migration out of the fibrin clot.

### Cytoskeletal and Antiosteocalcin Staining

Immunostaining was performed after each time point of culture. Briefly, cells were fixed by 4% paraformaldehyde solution for 30 min and incubated in a cold cytoskeleton buffer (3 mM MgCl_2_, 300 mM sucrose, and 0.5% Triton X-100 in PBS solution) for 5 min to permeabilize the membranes of the cells. The permeabilized cells were incubated in a blocking buffer solution, 5% fetal bovine serum (FBS), 0.1% Tween-20, and 0.02% sodium azide in PBS for 30 min. For antiosteocalcin, the dye was diluted in PBS (1:80) and incubated for 1 h at room temperature, followed by incubation with a secondary antibody conjugated with Alexa Fluor 488, 1:500 (green). For F-actin, rhodamine–phalloidin (1:300) was added to the cells for 1 h. Further, the cells were incubated in 4′,6-diamidino-2-phenylindole (DAPI) for 5 min to counterstain the nucleus. The fluorescent-dye-treated cells were observed and imaged using a laser scanning confocal microscope (Zeiss LSM 710 inverted confocal microscope, Germany).

### Alkaline Phosphatase Assay

Alkaline phosphatase (ALP) was measured after 1 and 2 weeks of culture using an alkaline phosphatase substrate kit. At the end of each culture time, the scaffolds were washed by PBS and cells were lysed using 1% Triton X-100. Then, 80 μL of the cell lysate mixture was added to 50 μL of the *para*-nitrophenylphosphate (*p*NPP) substrate (5 mM) and incubated at room temperature for 2 h. The reaction was inhibited by the addition of a stop solution, and the absorbance was measured at 405 nm using a multimode plate reader (PHERAstar FS, Germany).

### Alizarin Red Staining

After 14 days of culture, the media were removed and the cells were washed three times with PBS, fixed with 4% paraformaldehyde, and incubated for 15 min at room temperature. Detailed information is provided in the Experimental Section, Supporting Information.

### Quantitative Real-Time Polymerase Chain Reaction (RT-PCR)

The BM-MSCs were cultured on different scaffolds with an osteogenic medium for 4 weeks. Total RNA was extracted using the TRIzol reagent. RNA concentration and purity were measured using a NanoDrop 8000 spectrophotometer (Thermo Fisher). Complementary DNA (cDNA) was synthesized using the ImProm-II Reverse Transcription System. Primer sequences were taken from previously published studies and are summarized in Table 1. Relative quantification was performed using the comparative CT (2-ΔΔCT) and normalized against glyceraldehyde 3-phosphate dehydrogenase (GAPDH), which was used as a housekeeping gene to calculate the fold change in gene expression. BM-MSCs on a 2D culture using basal media were used as controls.

**Table 1:** Primers Used to Modify Bone-Specific Genes

gene	primer sequence
ALP	forward 5-GCACCTGCCTTACTAACTC-3
	reverse 5-AGACACCCATCCCATCTC-3
IBSP	F CACTGGAGCCAATGCAGAAGA
	R TGGTGGGGTTGTAGGTTCAA
BMP-2	F 5-TGCGGTCTCCTAAAGGTC-3
	R 5-AACTCGAACTCGCTCAGG-3
RUNX2	F TCAACGATCTGAGATTTGTGGG
	R GGGGAGGATTTGTGAAGACGG
osteopontin	F GAAGTTTCGCAGACCTGACAT
	R GTATGCACCATTCAACTCCTCG

### *In Vitro* Angiogenesis Study

Peptide hydrogel or collagen was placed in a 24-well plate, and human umbilical vein endothelial cells (HUVECs) at 40 000 cells/well were added on top of the peptide gel or collagen. Cells were cultured in endothelial growth media for 24 h. Cells were then investigated using an inverted microscope, and images were analyzed by ImageJ using the Angiogenesis Analyzer.

### Statistical Analysis

Results are represented as mean ± standard deviation (SD), *n* ≥ 3. The differences observed in the BM-MSC behavior between different scaffolds were compared; statistical analysis was performed using a Student *t*-test, and values with *p* < 0.05 were considered to be statistically significant.

## Results and Discussion

### Self-Assembling Ultrashort Tetrapeptides

Two self-assembling tetrapeptides, aromatic IVFK and nonaromatic IVZK, had been rationally designed based on a previous report of the positive impact of lysine (Lys, K) containing peptide hydrogels on cell expansion.^44,51^ The positively charged amine group from the lysine residue and the polarity of the surface have been reported to mediate cell adhesion and spreading.^52,53^ These peptides are composed of a positively charged amino acid (Lys) in the C-terminal and three nonpolar amino acids as a hydrophobic tail. Due to their amphiphilic structure, both peptides are able to self-assemble to form ordered aggregates.^52−54^ The aggregation rate of the peptides can be enhanced by alternating the aromatic phenylalanine (Phe, F) residue in IVFK with more hydrophobic, nonaromatic cyclohexylalanine (Cha, Z) as can be found in IVZK.^55^

Two-dimensional (2D) NMR experiments, such as correlation spectroscopy (COSY), total correlation spectroscopy (TOCSY), and nuclear Overhauser enhancement spectroscopy (NOESY), were then performed to analyze the spatial arrangement of the peptide molecule in water due to the self-assembly (Figures S3–S6 and Tables S1 and S2). We determined the intermolecular cross-peaks by eliminating the NOESY spectra that overlap with TOCSY spectra. Using this approach, we observed two nuclear Overhauser effect (NOE) signals from IVFK, in which one of them was assigned to the ε proton of Lys and the δ proton of the Ile. Another signal was arising from the interaction between β protons of phenylalanine with the γ proton of Ile. From these NOESY spectra, we proposed a formation of antiparallel configuration for IVFK. In addition, this antiparallel conformation was also predicted in IVZK as the amide proton of cyclohexylalanine interacts with the α proton of isoleucine (Ile, I). This result is congruent with the previous report on the formation of antiparallel conformation during the self-assembly of ultrashort peptides.^44,49^

**Figure 1:**
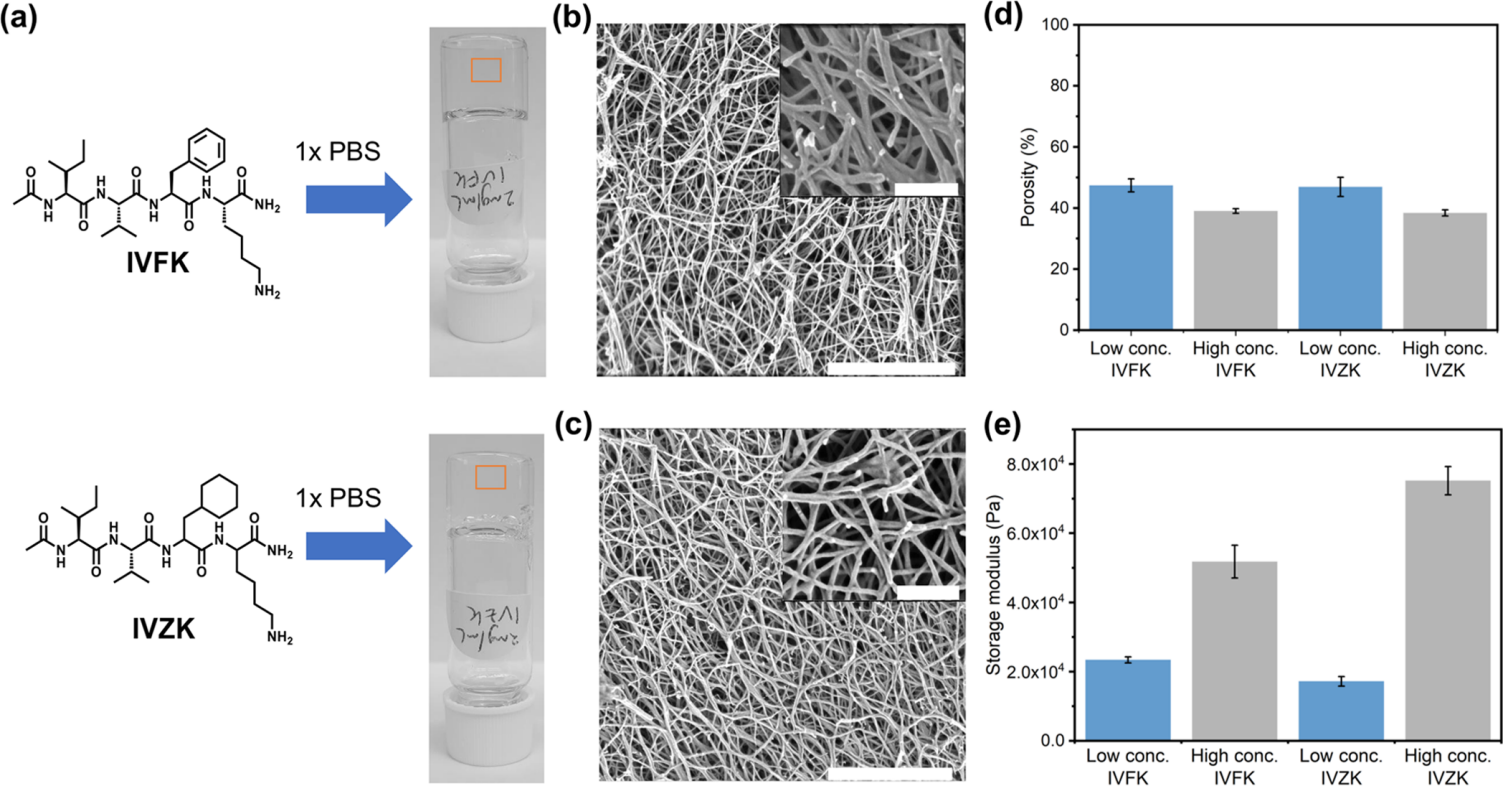
Self-assembling peptide hydrogels. The self-assembling peptides IVFK and IVZK at the critical gelation concentration (CGC) of 2 mg/mL generate supramolecular nanofibrous hydrogels in an aqueous solution. (a) The gelation was formed in 1× phosphate-buffered saline. The morphology of the nanofibrous hydrogels studied was imaged at magnifications of 100 000× and 500 000× (inset) using SEM: (b) 4 mg/mL IVFK and (c) 3 mg/mL IVZK. Scale bars are 1 μm and 200 nm (inset). (d) The porosity of the peptide hydrogels was calculated from the SEM images of both peptides at low concentrations (4 mg/mL IVFK and 3 mg/mL IVZK) and high concentrations (8 mg/mL for both peptides). (e) The mechanical stiffness values of the two peptide hydrogels IVFK and IVZK were measured at 1 rad/s and 0.1% strain.

Furthermore, we observed an instant hydrogel formation when PBS buffer was added to the peptide solution. To monitor the gelation time, a vial inversion test was performed at different peptide concentrations in 1× PBS to meet physiological conditions (Figures 1A and S7). Compared to IVFK, IVZK needed less time to form the hydrogel. This is most likely due to the presence of the highly hydrophobic cyclohexylalanine residue, which increases the aggregation rate.^44,56^

The morphology of the hydrogels was evaluated by performing SEM of dried hydrogels. The SEM micrographs confirmed the presence of porous fiber networks formed by the entanglement of self-assembled peptide nanofibers (Figures 1b,c and S8). This porous structure is vital to the diffusion of necessary nutrients for cell growth. We then compared the porosity of the hydrogels at low concentrations (4 mg/mL for IVFK and 3 mg/mL for IVZK) and high concentrations (8 mg/mL for both peptides). The results suggest that the increment of the peptide hydrogel reduced the porosity by 8% for both peptides (Figures 1d and S8). The dimensions of the pores (nanometer-scale) are far smaller than the cell’s nucleus dimensions, which might then restrict the passive cell movement. Under this condition, the cellular motility can be accommodated by either mechanically distorting the surrounding matrix^57^ or squeezing the cell morphology.^58−62^ It has been previously reported that the porosity of a planar matrix with a nanometer-scale did not significantly affect the differentiation of stem cells, but the stiffness of the matrix regulates the differentiation.^63^

### Mechanical Stiffness of Peptide Hydrogels

The mechanical stiffness of each peptide hydrogel was assessed by the storage modulus (*G*′) at different concentrations. As seen in Figure 1e and Table S3, the stiffness of the peptide hydrogels increases as the peptide concentration increases. The IVZK hydrogel showed a higher *G*′ value compared to IVFK at the same concentration, which is most likely due to the hydrophobic cyclohexylalanine residue in IVZK. Remarkably, the stiffness range of both peptide hydrogels was found to be within the range that supports multipotency maintenance.^51^ Therefore, these self-assembled peptide hydrogels were proposed to be promising candidates for use as cell-laden scaffolds in an osteogenic model.

### Viability, Attachment, and Proliferation of BM-MSCs in Scaffolds

**Figure 2:**
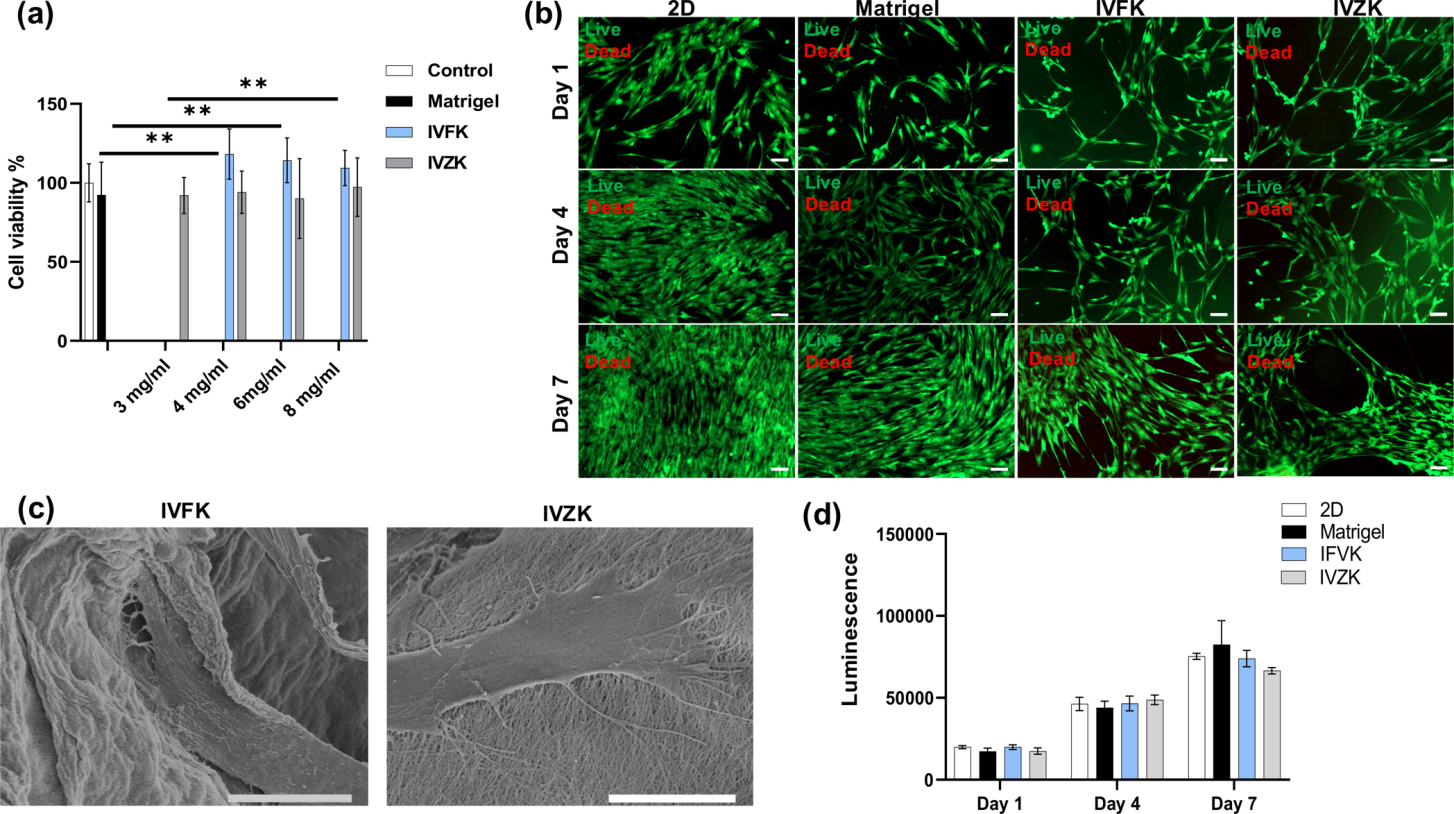
Cell biocompatibility, attachment, and proliferation. Cell viability as influenced by various concentrations of peptide solutions. The cells were incubated for 48 h with the peptides and without peptides as the control. (a) The cell viability was assessed using the alamarBlue assay. (b) Live/dead cell viability staining images of BM-MSCs within IVFK and IVZK peptide hydrogels. Cells were stained with calcein-AM (green, live cells) and ethidium homodimer-1 (red, dead cells). Scale bar is 100 μm. (c) SEM images of BM-MSCs in IVFK and IVZK; scale bars are 5 and 10 μm, respectively. (d) Three-dimensional cell viability assay of BM-MSCs in IVFK and IVZK after 1 and 7 days of culture.

After studying the inherent properties of the assembled peptide scaffolds, the hydrogels were screened for biocompatibility, cell attachment, and proliferation. Different concentrations of the peptides were used to study the biocompatibility of the cells within the constructs (Figure 2a). The results showed that no cytotoxicity effect was observed. Furthermore, IVFK showed a significant increase in cell growth compared to both controls (2D and Matrigel). Furthermore, a live/dead cytotoxicity assay was performed to evaluate the biocompatibility of MSCs in the peptides after different time points, as shown in Figure 2b. The results showed a high percentage of cell viability and increase in the cell growth rate during the culture time, thus indicating that there is no cytotoxicity associated with the peptides tested with the cells (Videos S1 and S2). Cell attachment and spreading into the hydrogels were observed within 24 h, 48 h, and 7 days, as shown in the bright-field microscopy images in Figure S9. The light micrographs showed long spindlelike cells spreading in all of the scaffolds tested. With culture time increasing, the cell growth increased in both peptides and the cells were observed to start spreading to form the spindle morphology. After 14 days in culture, most of the scaffolds were covered by cells (Figure S9). SEM images for MSCs cultured after 14 days in IVFK or IVZK showed clearly the elongated spindlelike morphology of the cells and the interaction between the cell’s filopodia and the matrix (Figure 2c). Furthermore, we also estimated the cell growth of MSCs in these peptide scaffolds by examining adenosine 5′-triphosphate (ATP) release after 1 and 7 days of culture (Figure 2d). ATP production increased with time of culture, indicating the proliferation of the cells among different peptides, which is comparable to the controls.

### Characterization of BM-MSCs in Scaffolds

To identify if the cells maintained their multipotency and capacity for self-renewal, MSCs were evaluated for two surface markers (CD73 and CD105) by flow cytometry. CD73 is a major cell surface marker defining MSCs. Interestingly, the CD73 expression is regulated by one of the main pathways in bone homeostasis.^64,65^ CD73 has recently been reported to have an important role in supporting osteogenic differentiation.^66^ Endoglin CD105 is another MSC marker that plays an important role in the processes of cell proliferation, differentiation, and migration. Gazit et al. demonstrated that CD105-positive MSCs are multipotent *in vitro* and can support bone formation *in vivo*.^67^ As such, we studied the CD105 and CD73 expressions through flow cytometry after 3 days of culture. In both scaffolds tested, the cells expressed their native CD105 and CD73, which suggests that the cells maintained their multipotency within the hydrogels (Figure S10). In addition, MSCs were entrapped in the fibrin clot and embedded within the gels to evaluate the ability of cells to migrate toward the surrounding environment (hydrogels). A previous study reported that cells failed to migrate to the hydrogel without RGD.^50^ Interestingly, cells were able to migrate radially out of the fibrin clot into hydrogels without any further functionalization and showed spindlelike shapes (Figure 4a). Confocal laser microscopy with nuclei and actin staining revealed clearly that the migration of these cells occurred.

**Figure 3:**
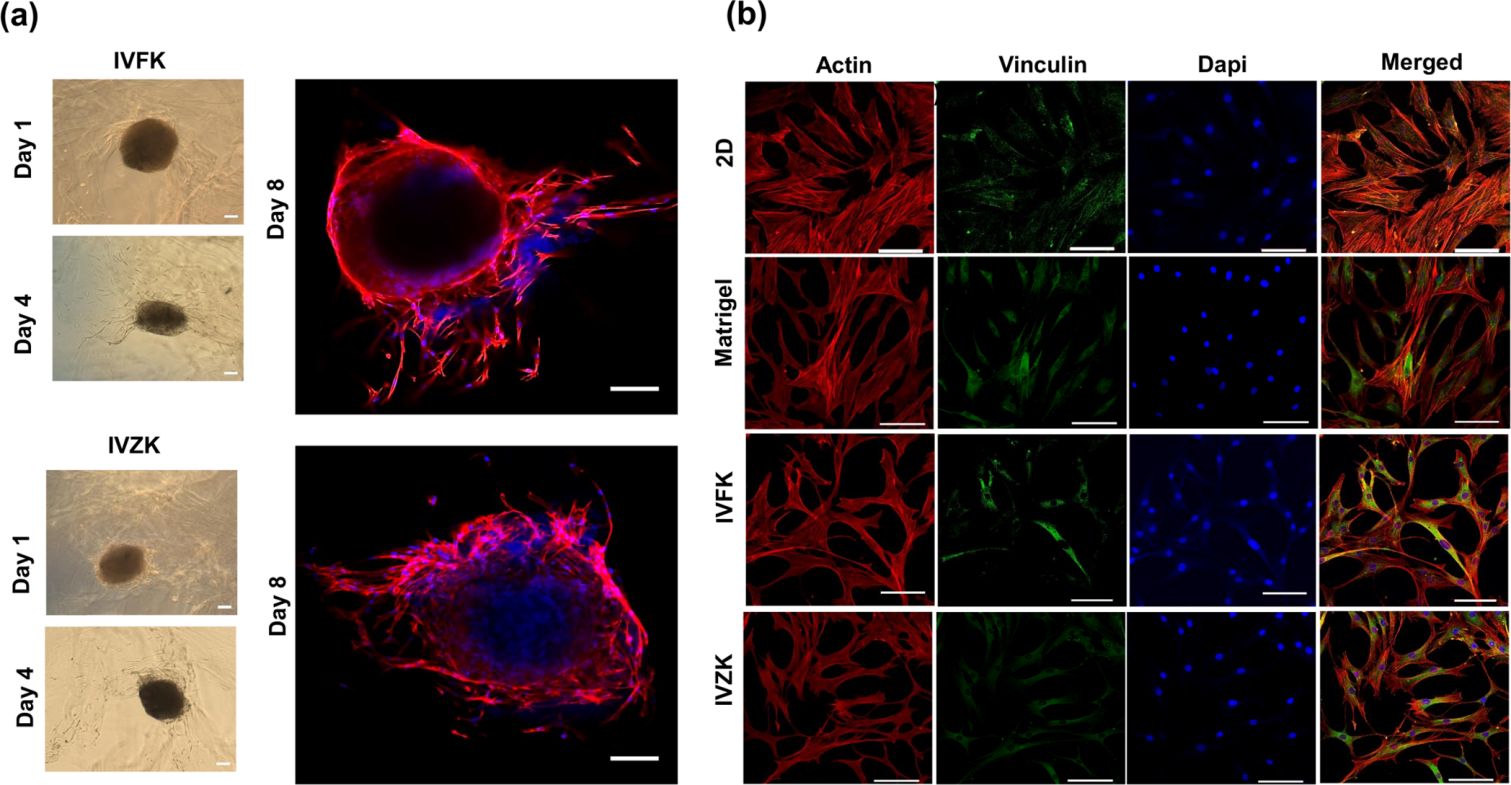
Characterization of BM-MSCs in scaffolds. (a) Migration assay: F-actin was stained with phalloidin (red) and the nucleus with DAPI (blue), scale bar is 100 μm. (b) Morphological studies of the BM-MSCs allowed for the assessment of their ability to develop well-organized actin cytoskeletons through actin immunostaining (red) and cell–matrix adhesion by stained vinculin (green) and DAPI for the nucleus (blue). Scale bar is 100 μm.

The extracellular matrix plays an essential role in several factors affecting a cell’s life such as proliferation and viability.^68^ In addition to differences in cell growth and viability, cells discriminate between matrices by controlling the level of tension in cell binding and then responding with counteracting forces. To further study how the cells responded to different scaffolds, immunostaining of the actin cytoskeleton was performed. Focal adhesions (FAs) act as force sensors between cells and their surrounding matrix through anchored actin microfilament bundles.^69,70^ As such, the cells were immunostained with F-actin using phalloidin to label the cytoskeletal arrangement. BM-MSCs were cultured in each peptide hydrogel studied earlier as well as in 2D culture and Matrigel as controls. The cells were able to attach to both scaffolds without any observed changes in their morphological appearance. The cells maintained their spindle morphology, spread in all directions, exhibited a meshlike/extended actin, and made a sheet of cells covering every part of scaffolds as shown in (Figure 3b).

Many studies have been performed on coated 2D surfaces that are not physiologically relevant,^71−73^ thus not providing an accurate reflection of the state of the cells. In contrast, 3D cultures may more closely mimic the natural cell environment and provide cells with the required stiffness conditions. One of these studies found that cells that grew on a 2D surface coated with collagen showed less actin cytoskeleton organization when compared to cells grown on a stiffer material.^74^ Furthermore, Tan et al. indicated that the cells cultured in stiff 3D matrices like transglutaminase cross-linked gelatin (TG-gel) with reported stiffnesses of 58 and 34 kPa formed dotlike actin filaments, and the cells did not spread through the scaffold.^75^ However, in this study, we found that all of the matrices supported a well-established cytoskeleton with an elongated arrangement (Figure 3b). One possible explanation for these observations is that in a 2D culture, cells are directed from the latitudinal direction only, thus allowing them the possibility to extend extremely along the longitudinal direction. On the other hand, the cells cultured in a 3D environment are grown in two directions, longitudinal and latitudinal.^75^ As such, the actin filaments of the cells in the 3D matrix showed better arrangement when compared to those grown in a 2D culture. Given that 3D environments provide an extra dimension for exterior mechanical responses and cell attachment, they can affect cell spreading, cell contraction, and intracellular signaling.^76,77^ Thus, the cells on 2D surfaces are less mechanically sensitive than those in 3D environments.^75^ Furthermore, the cells cultured in hydrogels were found to have a similar F-actin organization trend as in Matrigel.

Vinculin is an adhesion protein located in the cell–cell junctions and in focal adhesions (FAs), where it helps with the actin cytoskeleton connection to ECM.^64,65^ The effects of vinculin on the migratory behavior of cells in 3D collagen were reported. Deficiency in vinculin affected cell adhesion, contractility, and proliferation.^78^ The confocal fluorescence images of actin cytoskeleton and vinculin for the cells in IVFK, IVZK hydrogels, and Matrigel are shown in Figure 3b. In the 2D study, the cells appeared to be more spread out. However, in 3D culture, cells showed a smaller cell spreading area. While a previous report has shown that there is no enzymatic activity of vinculin, it can bind to actin, thereby activating actin polymerization.^65^ Most importantly, the distribution of vinculin was concentrated around the nuclear region, rather than being aligned with actin.^79−81^

### Osteogenic Differentiation of MSCs in Self-Assembled Peptides

The proliferation and attachment of MSCs were investigated in the three-dimensional network of various hydrogels. Next, we evaluated the ability of our hydrogels to support the osteogenic differentiation of MSCs. Cells were cultured in both hydrogels under the osteogenic condition for 3 weeks, and the efficiency of differentiation was compared to collagen. Collagen is an essential component of the ECM, which has been used widely as an important component of scaffolds in tissue engineering and is known to support both osteogenic differentiation of MSCs and angiogenesis of endothelial cells.^23^

**Figure 4:**
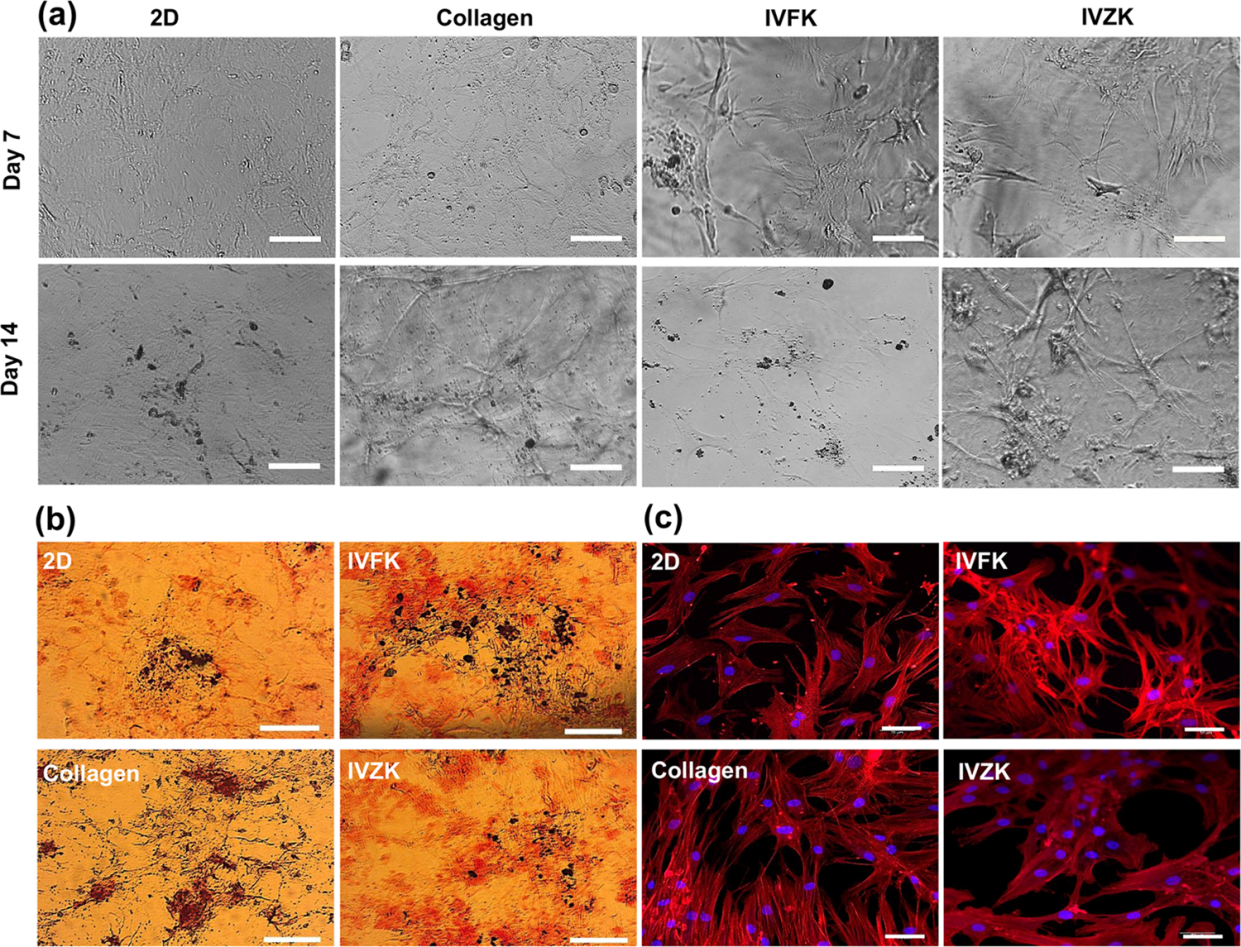
Osteogenic differentiation of MSCs in each scaffold. (a) Phase-contrast image of MSCs cultured in different hydrogels in osteogenic media after 7 and 14 days, which clearly showed the mineralization; scale bar is 100 μm. (b) Alizarin red-S staining of four scaffolds; scale bar is 100 μm. (c) *In vitro* morphology of hMSCs after 3 weeks of culturing. The cells displayed a highly branched “osteocyte-like” shape. Red represents the actin filaments, and blue represents the cell nuclei; scale bar is 50 μm.

The osteogenic differentiation potential of BM-MSCs in the hydrogels was observed, and bright-field images were taken after 7 and 14 days of culture. The morphology of cells changed several days after the addition of osteogenic induction media with the mineralization clearly observed as a black aggregate after 14 days. Also, MSCs exhibited a highly branched “osteocyte-like” shape, shown by bright-field images and more clearly by confocal fluorescence images of the F-actin cytoskeleton of the cells. This branched shape is correlated with the differentiation of stem cells toward an osteogenic lineage, which indicates that the cells successfully differentiated in both scaffolds (Figures 4a,c and S11a).^82−84^

Alizarin red staining (ARS) was used to detect calcium deposition. The BM-MSCs cultured in the different scaffolds in the osteogenic medium were stained by Alizarin red to confirm the mineralization process during osteogenic differentiation (Figures 4b and S11b). There were detectable mineral deposits, which are seen as small, stained nodules in dark-red/black color. This indicates the presence of calcium deposits. The mineral produced by the cells cultured in the IVFK hydrogel in the osteogenic medium produced the most intense ARS staining when compared to the other scaffolds tested. These results showed that IVFK could accelerate the production of calcium and regulate the calcification progression of the bone matrix.

**Figure 5:**
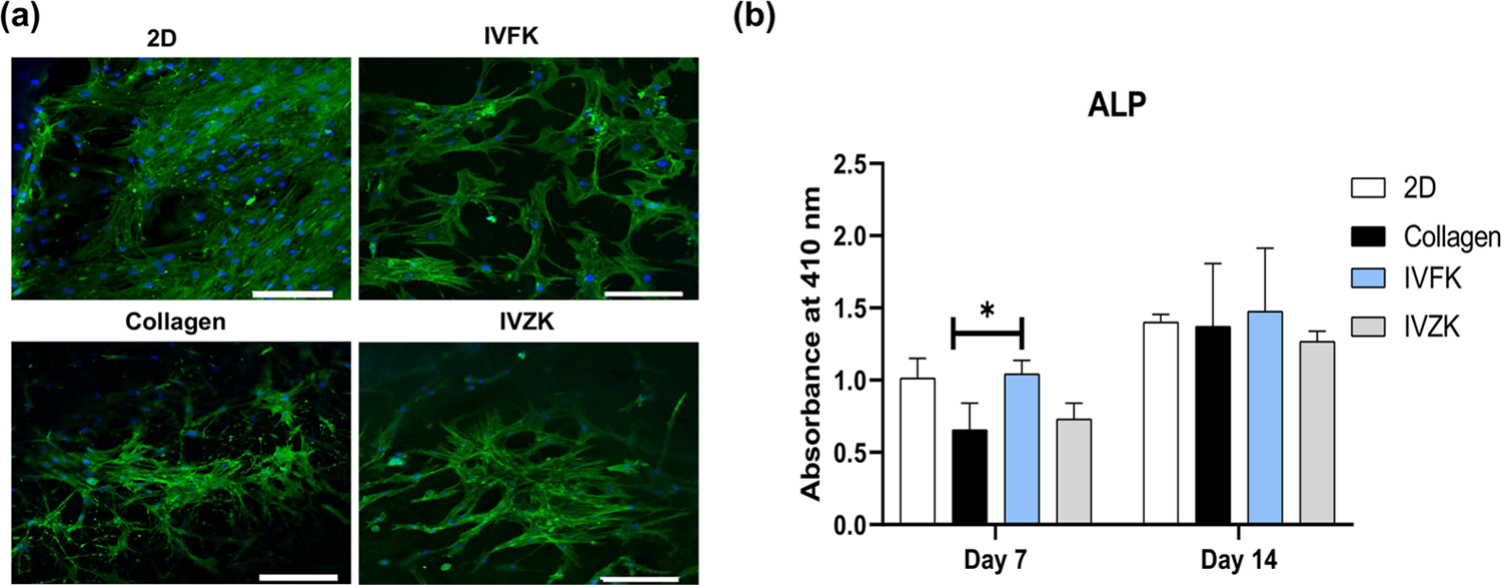
Osteogenic differentiation of BM-MSCs in scaffolds. (a) Confocal images showed the expression of osteocalcin in green, and the cell nuclei were stained using DAPI; scale bar is 100 μm. (b) ALP activity of BM-MSCs cultured on scaffolds in the osteogenic medium for 7 and 14 days. All values are expressed as mean ± SD from three different replicates. Statistical significance of *p < 0.05.

Further confirmation was done to prove the differentiation of BM-MSCs to the osteogenic lineage; the expression of osteocalcin was stained and imaged after 3 weeks of culture using confocal microscopy (Figure 5a). While the MSCs cultured in both scaffolds were able to express osteocalcin, the expression levels in IVFK were comparable to those cultured in the collagen control group. Additionally, the levels of alkaline phosphatase (ALP), an early osteogenic marker expressed by osteoblast cells, were measured to confirm the commitment of BM-MSCs toward the osteogenic lineage. The results below (Figure 5b) show the time course of ALP activity in MSCs cultured on 2D and in different scaffolds: collagen, IVFK, and IVZK after 7 and 14 days. Significantly higher ALP activity was detected for MSCs cultured in the IVFK hydrogel than in those cultured in collagen after 7 days (*p* < 0.05). Furthermore, the ALP activity increased with time during the initial 2 weeks as an indicator of osteogenic differentiation.

### Gene Expression Analysis

**Figure 6:**
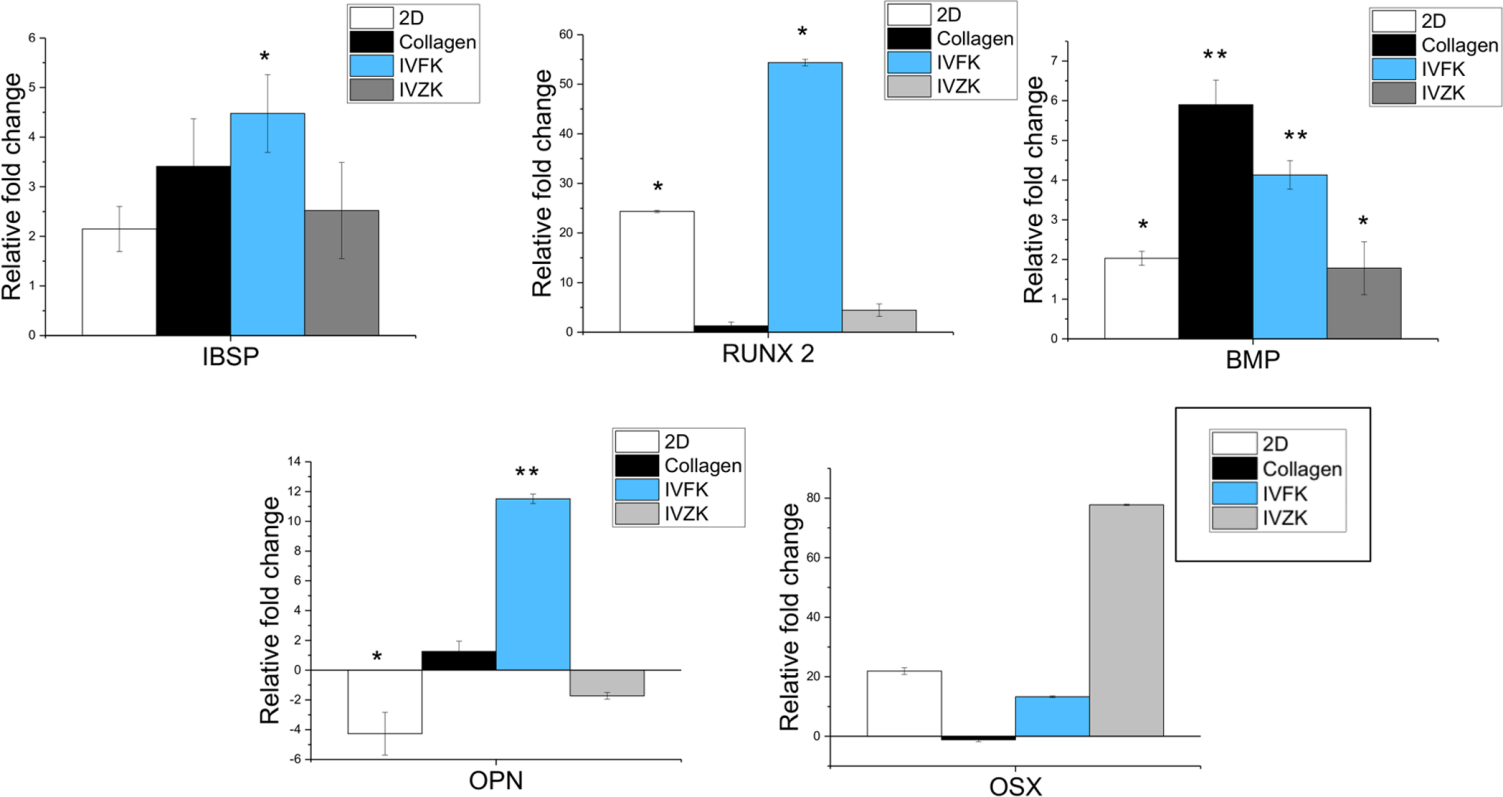
Osteogenic-related gene expression of BM-MSCs cultured on different scaffolds after 30 days. Data analysis and relative quantitation were performed using the comparative CT (ΔΔCT) method. Statistical significance of *p < 0.05 and **p < 0.001.

Real-time polymerase chain reaction (PCR) values of the BM-MSC gene expressions of the bone morphogenetic protein (BMP-2), bone sialoprotein 2 (IBSP), osteopontin (OPN), osterix (OSX), and RUNX2 were evaluated after 30 days of culture (Figure 6). Runx2 is an early marker of osteogenic differentiation.^85^ The increase in Runx2^86^ indicates that the MSCs were being directed toward the bone lineage.^1^ The expression of Runx2 was higher in IVFK when compared to cells cultured in other groups.

BMP-2 is a glycoprotein that is responsible for the differentiation of osteoblasts, thereby helping in bone formation.^87^ It is known that the expression of BMP-2 is upregulated in hMSCs during osteogenic differentiation.^88,89^ The expression was upregulated in both peptide scaffolds as well as in collagen and 2D.

OPN and IBSP are considered as late osteogenic markers, and their expression is known to be increased to induce osteoblast differentiation toward mature osteocyte.^90^ Osteopontin (OPN) is one of the most plentiful noncollagenous proteins in the bone. OPN plays an important role in differentiating osteoclasts and in recruiting and functioning osteoblasts.^91^ OPN was also found to help in osteoclast migration toward sites of resorption and is essential for normal resorption and bone turnover.^92^ The expression of this gene was measured and found to be downregulated in 2D and IVZK hydrogels. However, importantly, the expression of ONP was upregulated in cells cultured in IVFK as well as in collagen, which indicates the maturation of osteocyte. In addition, the IBSP gene expression level was found to be the highest in the IVFK scaffold. Finally, we found that the expression level of OSX, which is decreased in the final maturation phase of osteogenic differentiation,^90^ was low in IVFK compared to other groups and downregulated in cells cultured in collagen. These findings clearly point to the osteogenic differentiation advantages offered by the IVFK peptide hydrogel.

### Effect of Matrix Stiffness on Osteogenic Differentiation

Cells are sensitive to many factors, which may, in turn, affect their growth, maturation, and differentiation. These factors include chemical stimuli like growth factors and other factors.^93−96^ Furthermore, the mechanical properties of the extracellular matrix, like rigidity, elastic modulus, and porosity, also have a significant impact.^97−99^ Mechanical properties have been reported to have a substantial effect on regulating the stem cell fate.^100^ For example, cells cultured inside hydrogel scaffolds with elastic moduli in ranges of 11–30 and 2.5–5 kPa directed MSC differentiation into osteogenic and adipogenic lineages, respectively.^101^

**Figure 7:**
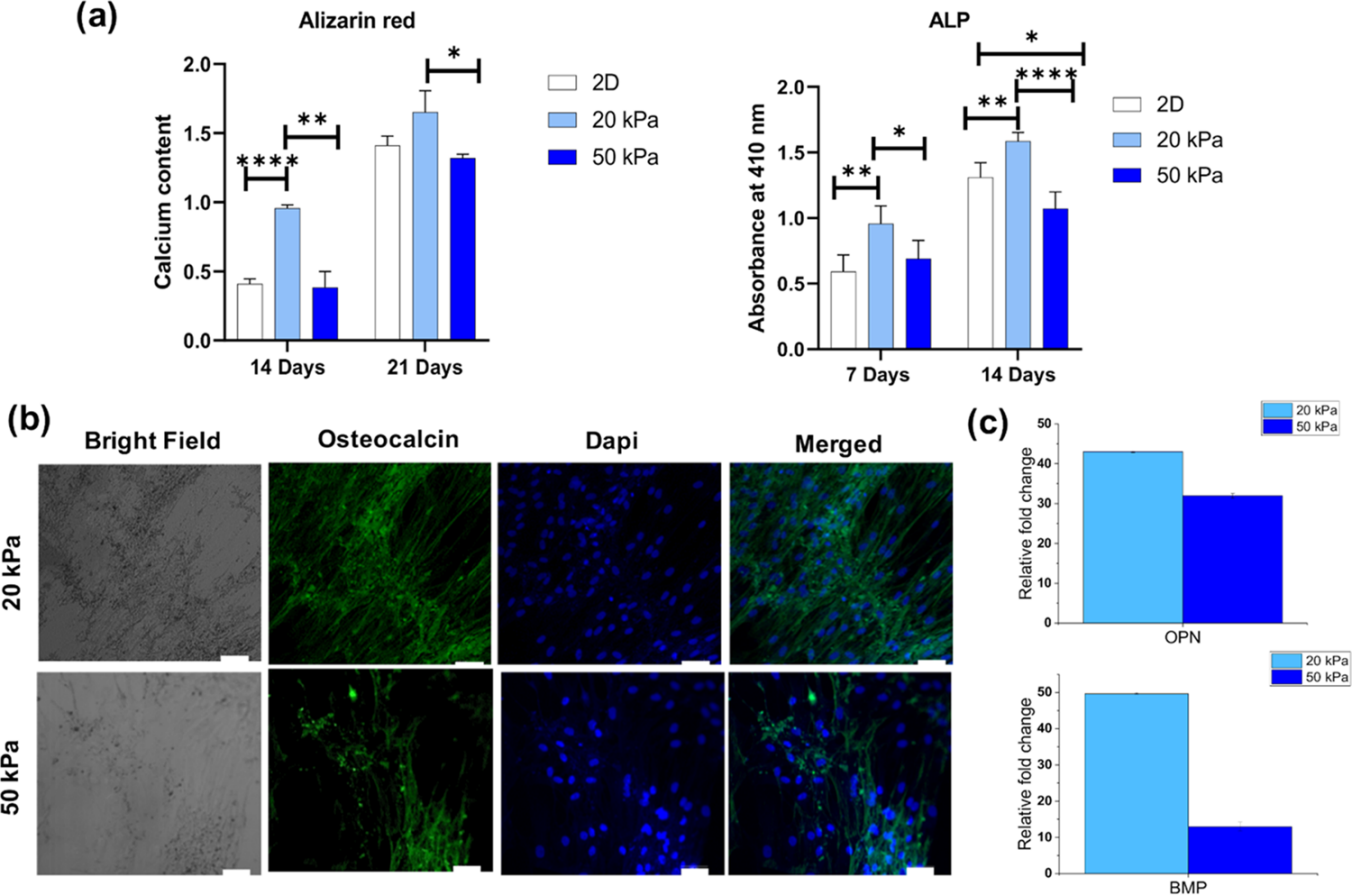
Osteogenic differentiation of BM-MSCs in the IVFK hydrogel with two different stiffnesses (20 and 50 kPa). (a) Quantification of mineralization by Alizarin red-S after 14 and 21 days of culture and ALP activity of BM-MSCs after 7 and 14 days. (b) Confocal images of osteocalcin produced by differentiated cells in different scaffolds; the cell nuclei were stained using DAPI. (c) Osteogenic-related gene expression by RT-PCR of BM-MSCs cultured within scaffolds with different stiffnesses. All values are expressed as mean ± SD from three different replicates. Statistical significance of *p < 0.05, **p < 0.01, and ****p < 0.0001.

Based on the cell attachment, proliferation, and calcium deposition results obtained earlier, we selected the IVFK scaffold to be used in the subsequent bone differentiation studies. Therefore, osteogenic differentiation of BM-MSCs in IVFK hydrogels of different stiffnesses was evaluated using the alkaline phosphatase (ALP) activity assay, Alizarin red-S staining, and osteocalcin staining and by measurement of osteogenic transcription levels (Figure 7).

The mechanical properties of the peptide hydrogel can easily be tuned by increasing the peptide concentration. However, caution should be administered as increasing the peptide concentration will affect the peptide hydrogel’s physical parameters, such as porosity and diffusion of nutrients, affecting the cell viability and proliferation.^102−104^ To study the effect of peptide hydrogel stiffness on MSC differentiation, two concentrations of IVFK were investigated. Peptide concentrations of 4 and 8 mg/mL resulted in stiffnesses of around 20 and 50 kPa, respectively. Analyses of the calcium content and ALP release after 7 and 14 days, respectively, revealed that the value of the hydrogel with a storage modulus of around 20 kPa possessed a significantly higher calcium content and ALP release than that at 50 kPa (Figure 7a). The osteocalcin expression was found to be more intense in the 20 kPa scaffold compared to the others (Figure 7b). Finally, the expression of two osteogenic genes showed that the hydrogel with a reported stiffness of 20 kPa was able to support cell differentiation much better than the 50 kPa scaffold (Figure 7c). This result is consistent with that of previous studies that reported that stiff matrices (16–25 kPa) lead MSCs to be differentiated to the osteoblast.^105,106^ Also, another group reported that cells cultured inside a hydrogel with stiffness 11–30 kPa directed MSC differentiation into the osteogenic lineage.^101^ The results suggest that matrix stiffness plays an important role in cell differentiation.

### Angiogenesis Ability *In Vitro*

**Figure 8:**
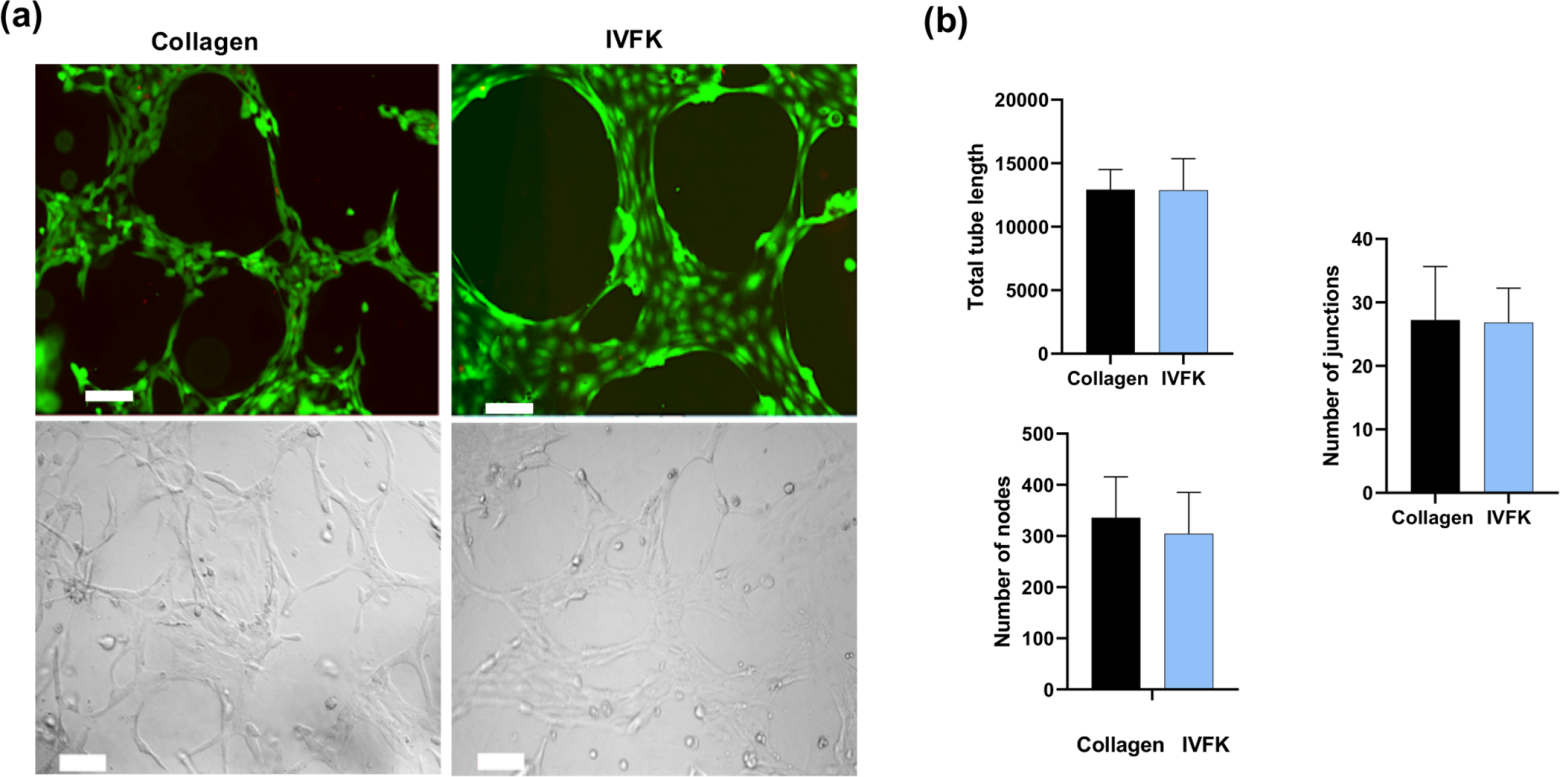
Angiogenesis ability of the IVFK scaffold *in vitro*. (a) Calcein-AM (green) and ethidium homodimer (red) staining, and bright-field image of HUVECs after 24 h; scale bar is 100 μm. (b) Quantification of angiogenesis by measuring vessel junctions, number of nodes, and the total vessel length for five different pictures.

When bone deficiency occurs, it often causes blood vessel damage. Blood vessels provide the necessary components to repair the region of bone defects by transferring oxygen and nutrients.^107,108^ Because the capillary system is a crucial component in bone regeneration, the capability of IVFK and IVZK hydrogels (Figure S12) to support angiogenesis was also evaluated using HUVECs and compared with collagen, which is an essential component of the ECM and is known to support the angiogenesis of endothelial cells.^23^ As indicated in Figure 8a, after 24 h of culture, HUVECs were observed under the microscope, and dense network structures were found in HUVECs cultured in the hydrogel similar to the collagen control. Additionally, the junctions, nodes, and the total length of tubes were analyzed by ImageJ, as shown in Figure 8b. No significant difference was found between IVFK and the positive control. This result indicates that the IVFK scaffold has the ability to promote angiogenesis of HUVECs *in vitro*.

## Conclusions

This work provides evidence of the successful preparation of an ultrashort, amphiphilic peptide hydrogel capable of promoting osteogenic differentiation and angiogenesis. In contrast with the traditional 2D cell culture, cells maintained in a 3D culture more closely mimic the *in vivo* setting. This is particularly true with respect to cell shape and organization, as well as the extracellular environment, which may have a substantial impact on cell behavior. This study aimed to investigate the potential of two peptide scaffold materials in supporting the adhesion, proliferation, and osteogenic differentiation of BM-MSCs. These hydrogels are easy to prepare and solidify quickly to provide a 3D environment. Furthermore, they have good mechanical properties, and they provide a well-defined molecule that can be adapted to include a wide range of chemical moieties. The fiber networks of the two hydrogels were found to resemble that of the native ECM while providing adhesion and proliferation cues for the BM-MSCs. Our hydrogels were biocompatible and promoted cell migration, osteogenic differentiation, and angiogenesis. Cells cultured in the IVFK hydrogel showed an increase in ALP production, an enhanced expression of osteogenic markers, and mineralization. Furthermore, the mechanical properties of the hydrogel can be modulated by changing the peptide concentration, which was found to influence cell behavior as well. Thus, our hydrogel supports both osteogenic differentiation and angiogenesis and could potentially be used as a scaffold in bone tissue engineering. Importantly, it provides valuable insights into the design of hydrogels for 3D stem cell culture in the future.

## Supporting Information Available

Materials and methods; liquid chromatograms and mass spectra of IVFK and IVZK (Figures S1 and S2); ^1^H NMR spectra of IVFK and IVZK in *d*_6_-dimethyl sulfoxide (*d*_6_-DMSO) (Figure S3); 2D NMR (COSY, TOCSY, NOESY) spectra of IVFK and IVZK in H_2_O/D_2_O 9:1 (Figures S4–S6 and Tables S1 and S2); vial inversion test for both IVFK and IVZK at different concentrations (Figure S7); porosity of peptide hydrogels (Figure S8); rheological properties of peptide hydrogels at different concentrations (Table S3); phase-contrast images of BM-MSCs cultured in different scaffolds up to 14 days (Figure S9); characterization of BM-MSCs in scaffolds; BM-MSCs stained against CD73 and CD105; data showing the positive expression of MSC-associated surface antigens CD73 and CD105 (Figure S10); BM-MSCs cultured in normal basal media (Figure S11); and angiogenesis ability of the IVZK scaffold *in vitro* (Figure S12) (PDF)
BM-MSCs cultured within IVFK for 4 days (Video S1) (AVI)
BM-MSCs cultured within IVZK for 4 days (Video S2) (AVI)

## Supplementary Material

bm1c00205_si_001.avi

bm1c00205_si_002.avi
