# Supplementary material for: Scaffolds from Self-Assembling Tetrapeptides Support 3D Spreading, Osteogenic Differentiation, and Angiogenesis of Mesenchymal Stem Cells
Source: Biomacromolecules. 2021 Apr 28;22(5):2094–106. doi: 10.1021/acs.biomac.1c00205 (PMC8382244; doi:10.1021/acs.biomac.1c00205)
Supplement: Supplementary file 3 — bm1c00205_si_003.pdf [file bm1c00205_si_003.pdf]

SUPPORTING INFORMATION

Scaffolds from Self-Assembling Tetrapeptides Support 3D Spreading, Osteogenic Differentiation and Angiogenesis of Mesenchymal Stem Cells

Salwa Alshehri, Hepi H. Susapto, Charlotte A. E. Hauser[*]

Laboratory for Nanomedicine, Division of Biological and Environmental Science and Engineering, King Abdullah University of Science and Technology, Thuwal, Kingdom of Saudi Arabia

*Corresponding author, E-mail: charlotte.hauser@kaust.edu.sa

EXPERIMENTAL SECTION

1. Materials S3
2. Peptide Synthesis S4
3. Liquid Chromatography – Mass Spectroscopy (LC-MS) S4
4. 1D NMR and 2D NMR in D_2O-H_2O S5
5. Scanning Electron Microscopy (SEM) S5
6. Alamar Blue S6
7. Live/Dead assay S6
8. 3D Cell Proliferation Assay S7
9. Flow Cytometry S7
10. Alizarin Red Staining S7

RESULTS

1. Figure S1. LC-MS spectra of IVFK S8
2. Figure S2. LC-MS spectra of IVZK S9
3. ^{1}H-NMR multiplet report S10
4. Figure S3. 2D NMR of IVFK in 90% H_2O - 10%D_2O mixture S11
5. Table S1: 1H-NMR chemical shifts of IVFK S12
6. Figure S4. 2D NMR of IVZK in 90% H_2O - 10%D_2O mixture S13
7. Table S2: 1H-NMR chemical shifts of IVZK S14
8. Figure S5. Antiparallel configuration of IVFK S15
9. Figure S6. Antiparallel configuration of IVZK S16
10. Figure S7. Vial inversion test S17
11. Figure S8. The porosity of peptide hydrogels at low and high concentration S18
12. Table S3. Rheological properties of peptide hydrogels at different concentrations S19
13. Figure S9. Phase-contrast imaging of BM-MSCs cultured in different scaffolds S20
14. Figure S10. Characterization of BM-MSCs in scaffolds. BM-MSCs were stained against CD73 and CD105 S21
15. Figure S11. BM-MSCs cultured in normal basal media S22
16. Figure S12. Angiogenesis ability of IVZK scaffold in vitro S23

REFERENCES S24

EXPERIMENTAL SECTION

Materials. MBHA Rink Amide resin, 9-fluorenylmethoxycarbonyl (Fmoc), and N,N,N′,N′-tetramethyl-O-(benzotriazol-1-yl)uronium tetrafluoroborate (TBTU), hydroxy benzotriazole (HOBt) were purchased from GL Biochem, China. Dimethylformamide (DMF), dichloromethane (DCM), N,N-diisopropylethylamine (DIPEA), piperidine, acetic anhydride, trifluoroacetic acid (TFA), triisopropylsilane, diethyl ether, and ethanol were purchased from Sigma-Aldrich. The chemicals were used as received, without any purification.

Bone marrow-derived mesenchymal stem cells (BM-MSCs, PT-2501) were purchased from Lonza, USA. Cells were cultured in medium (PT-4106E Lonza, USA) and supplemented with mesenchymal cell growth supplements (PT-4106E Lonza, USA), Gentamicin Sulfate Amphotericin-B (PT-4501E Lonza, USA) and with L-Glutamine (PT-4107E Lonza, USA). T175 or T75 cell culture flasks and 96 and 48 well-plates were ordered from Corning, USA. The CellTiter-Glo® luminescent 3D cell viability assay and TRIzol™ reagent (Invitrogen USA) ImProm-II™ Reverse Transcription System were purchased from Promega, USA. APC anti-human CD105 Antibody 43A3 and PE anti-mouse CD73 Antibody TY/11.8 were from BioLegend, UK. Anti-osteocalcin was purchased from (Abcam, USA). Alexa Fluor 488 (Invitrogen, USA), ALP kit (Abcam, UK). LIVE/DEAD Cell Viability Assay, Actin Cytoskeleton / Focal Adhesion Staining Kit were purchased from Thermo Fisher Scientific, USA, and Merck, Germany respectively. Alizarin red S dye was from Abcam,USA.

Peptide Synthesis. The rink amide resin (1 mmol) was pre-swollen in DCM for 30 min and the Fmoc-protecting group on the resin was then deprotected by 20% (v/v) piperidine/DMF prior to the first coupling of amino acid. Then, the resin was washed throughly with DMF and DCM. The peptide coupling was conducted on rink amide resin by adding a mixture of TBTU (3eq.), HOBt (3eq.)

DIPEA (6 eq.), and Fmoc-protected amino acid (3eq.) to the resin. Kaiser test was performed at the end of coupling step to confirm the attachment of amino acid. All the steps of Fmoc-deprotection, peptide coupling, washing and Kaiser test were repeated until all of the amino acids were added to the peptide sequence. The N-terminal of peptide sequence was later capped with acetyl group by adding a mixture of 2:6:1 (v/v) acetic anhydride:DIPEA:DMF. Then, the peptide was cleaved from the resin by agitating it in 95:2.5:2.5 mixture of TFA, water and triisopropylsilane, respectively for a minimum 2 hrs. The peptide in TFA solution was later collected in a round bottom flask. Afterwards, the peptide was precipitated by adding cold diethyl ether and kept standing overnight at 4°C. The precipitated peptide was separated from the supernatant by centrifugation and then dried under vacuum. This crude peptide was purified by reverse phase-HPLC purification using C-18 column (2-98 % ACN in 10 min) at the flow rate of 20 mL/min.

Liquid Chromatography – Mass Spectroscopy (LC-MS). 1 mg of peptides, which were dissolved in 1 mL of water were analyzed using Agilent 1260 Infinity LC equipped with Agilent 6130 Quadrupole MS. Agilent Zorbax SB-C18 4.6 x 250 mm column was used together with a mixture of two different solutions of 0.1% (v/v) formic acid – water (A) and 0.1% (v/v) formic acid – acetonitrile (B). The flow of mobile phase was 1.5 mL/min with a composition of 98% A – 2% B at first 1 min. From 1 to 18 mins, the flow of B increased until 98% B and turned back again to 2% again. LC chromatogram was obtained at wavelength of 220 nm. The molecular weight of the peptides was confirmed in positive mode polarity.

1D NMR and 2D NMR in D_2O-H_2O. 2D NMR of peptide solution was carried out to study the spatial structure during the assembly process. These NMR spectra were recorded on a Bruker Avance III 600 MHz equipped with a 5 mm Z-gradient SmartProbe BB(F)-H-D (BrukerBioSpin, Rheinstetten, Germany). The samples were prepared from 10 mg of peptide in a mixture of 900 µL of 1mM 2,2-Dimethyl-2-silapentane-5-sulfonate (DSS) and 100 µL of D_2O. ^{1}H-NMR was recorded

by collecting 32 scans using excitation sculping with gradients pulse program (zgesgp) for water suppression.[1] COSY and TOCSY were then conducted to see the correlation between hydrogens that were coupled to each other. COSY was performed using a time domain of 4096 (F2) × 512 (F1), 16 number of scans, a pulse program of cosydfgpph19, and 3-9-19 pulse sequence for water suppression.[2-4] For TOCSY, we set the parameters with time domain of 8192 (F2) × 512 (F1), 16 number of scans, a pulse program of dipsi2gpph19, and 3-9-19 pulse sequence for water suppression. Finally, the NOESY acquisition was carried out with a time-domain size of 2048 (F2) × 512 (F1), 32 scan numbers, a pulse program of noesyesgpph19, a mixing time of 400 ms, and water suppression using 3-9-19 pulse sequence. Bruker Topspin 3.5pI7 software was used in all NMR experiments for both data collection and spectral analyses.

Scanning Electron Microscopy (SEM). Peptide hydrogels were characterized using SEM to visualize the morphology of the nanofibers. In the absence of cells, 50 μL of both peptide hydrogels at low (4 mg/mL IVFK and 3 mg/mL IVZK) and high concentration (8 mg/mL for both peptides) were prepared on 18x18mm glass coverslips and left to solidify for 10-20 minutes post-formation. At this point, the hydrogel samples were dehydrated by gradually immersing in increasing concentrations of 20%, 40%, 60%, 80%, and 100% (v/v) ethanol solutions for 5 min in each ethanol solution. Further dehydration in 100% ethanol solution was done for 2 hours. The dehydrated samples were subsequently placed into a critical point dryer for evaporation before being mounted onto aluminum SEM pin stubs with double-stick conductive carbon tape. A final sputter coating of 10 nm of Iridium (Ir) was performed prior to imaging with FEI Magellan XHR. The porosity of the peptide matrix was analyzed using thresholding technique in *ImageJ*.

SEM was also used to determine the cell-matrix interaction. First, the cells encapsulated in hydrogels were fixed in 2.5% glutaraldehyde for overnight (4 °C) and then washed using PBS.

The samples were subsequently incubated in 1% osmium tetroxide for one hour and washed with water. Afterwards, the dehydration was done by immersing them in increasing concentrations of ethanol: 30, 50, 70, 90 and 100%, 15 min each step. Then, the samples were processed in critical point dryer. The dried samples were then mounted and coated with 5nm Ir thickness.

Alamar Blue. A volume of 10 μL of Alamar blue stock solution was added to 100 μL of media in each well. Plates were incubated at 37 C° for 4 h. The fluorescence was measured at an excitation wavelength of 530 nm, and the emission at 590 nm. The viability percentage was calculated against the control (2D).

Live/Dead assay. The viability of the cells within different scaffolds (IVFK 3mg/ml and IVZK 4 mg/ml) were tested using live/dead staining. In which, calcein acetoxymethyl ester (Calcein-AM) is used to detect viable cells and ethidium homodimer-I (EthD-I) is used to detect dead cells. 3D constructs were washed twice with PBS. Then a staining solution of 2 μM of Calcein-AM and 4 μM of EthD-1 were added toeach well and incubated for 30 minutes at room temperature. After the incubation period, the staining solution was discarded, and 1x DPBS was added to each well before imaging. Stained cells were imaged with an inverted confocal microscope (Zeiss LSM 710 Inverted Confocal Microscope, Germany).

3D Cell Proliferation Assay. The CellTiter-Glo® luminescent 3D cell viability assay was used to determine the proliferation of cells in 3D hydrogels based on the presence of ATP, as a product produced from metabolically active cells.[5] After each time point, the kit was equilibrated at room temperature for approximately 30 minutes. CellTiter-Glo® Reagent equal to the cell culture medium existing volume in each well was added. The contents were mixed for 5 minutes to digest the hydrogels and then incubated for 30 minutes at room temperature. After incubation, the luminescence was recorded using a plate reader (PHERAstar FS, Germany).

Flow Cytometry. The cells were cultured in different peptide scaffolds as described before. After 3 days of culture, the gels were transferred into a 15 mL centrifuge tube, 10 mL of PBS was added, and the hydrogels were mechanically disrupted by gently pipetting the mixture. Then the tube was centrifuged at 1,000 × *g* for 5 min, and the supernatant was discarded. The cells were resuspended in 0.5mL of Cell Staining Buffer including 5 µL of the following conjugated monoclonal antibody, APC anti-human CD105 Antibody, 43A3 and PE anti-human CD73 Antibody. The cells were incubating in the dark for 15 min with combinations of the monoclonal antibodies described above. Unstained hMSC was used as a control.

Alizarin Red Staining. After 14 days of culture, the media was removed and then, the cells were washed three times with PBS and then fixed with 4% paraformaldehyde and the cells incubated for 15 minutes at room temperature. Then, the cells were washed three times with MilliQ water. Then the MilliQ water was removed and 1 mL of 40 mM Alizarin Red Staining (pH= 4.2) was added to each well. The scaffolds were incubated at room temperature for 20-30 min with gentle shaking. Finally, the wells were washed five times with MilliQ water and imaged using an inverted microscope (Leica DM1300M). For the quantification of Alizarin Red Staining, 100 µL of 10% acetic acid was added to each well in a 48-well plate and incubated at room temperature for 30 minutes with gentle shaking. The samples were transferred into a 1.5mL microcentrifuge tube, and the tubes were vortexed for 30 seconds. The samples were heated at 85 °C for 10 minutes and sealed with parafilm to avoid evaporation. The tubes were then incubated on ice for 5 minutes and then centrifuged at 20,000g for 15 minutes. After that, 50 µL of 10% ammonium hydroxide was added to neutralize the acidic environment. Finally, 50 µL of each tube was transferred to a 96-well plate, and the absorbance at 405 nm was read with a plate reader (PHERAstar FS, Germany)

RESULTS

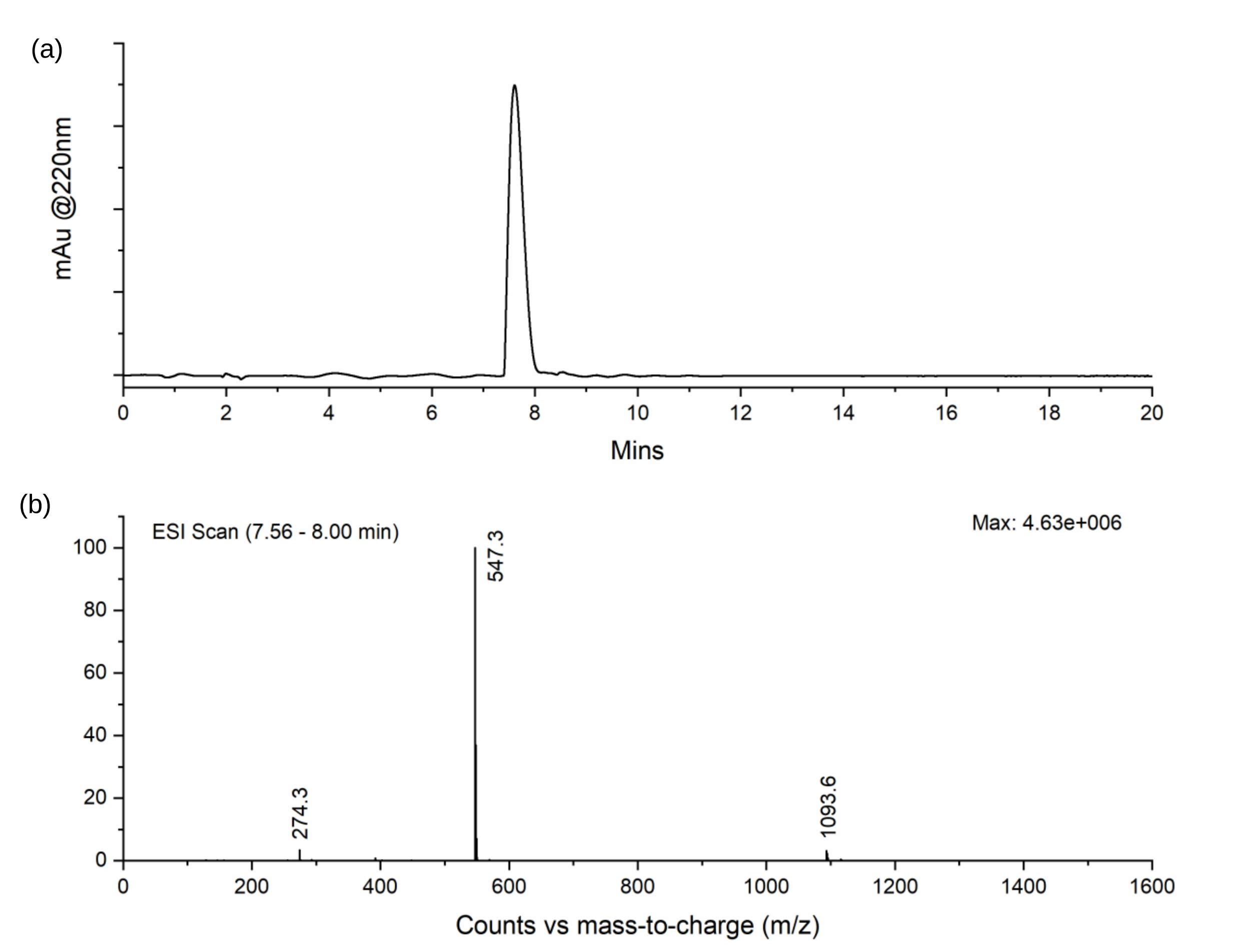

Figure S1. Characterization of IVFK by using LC-MS. a, Liquid chromatogram of IVFK by the absorbance at 220 nm, and b, mass spectrum of IVFK. MS: (m/z) calculated 546.7, $[M+2H]^{2+}$ found 274.3, $[M+H]^{+}$ found 547.3, and $[2M+H]^{+}$ found 1093.6.

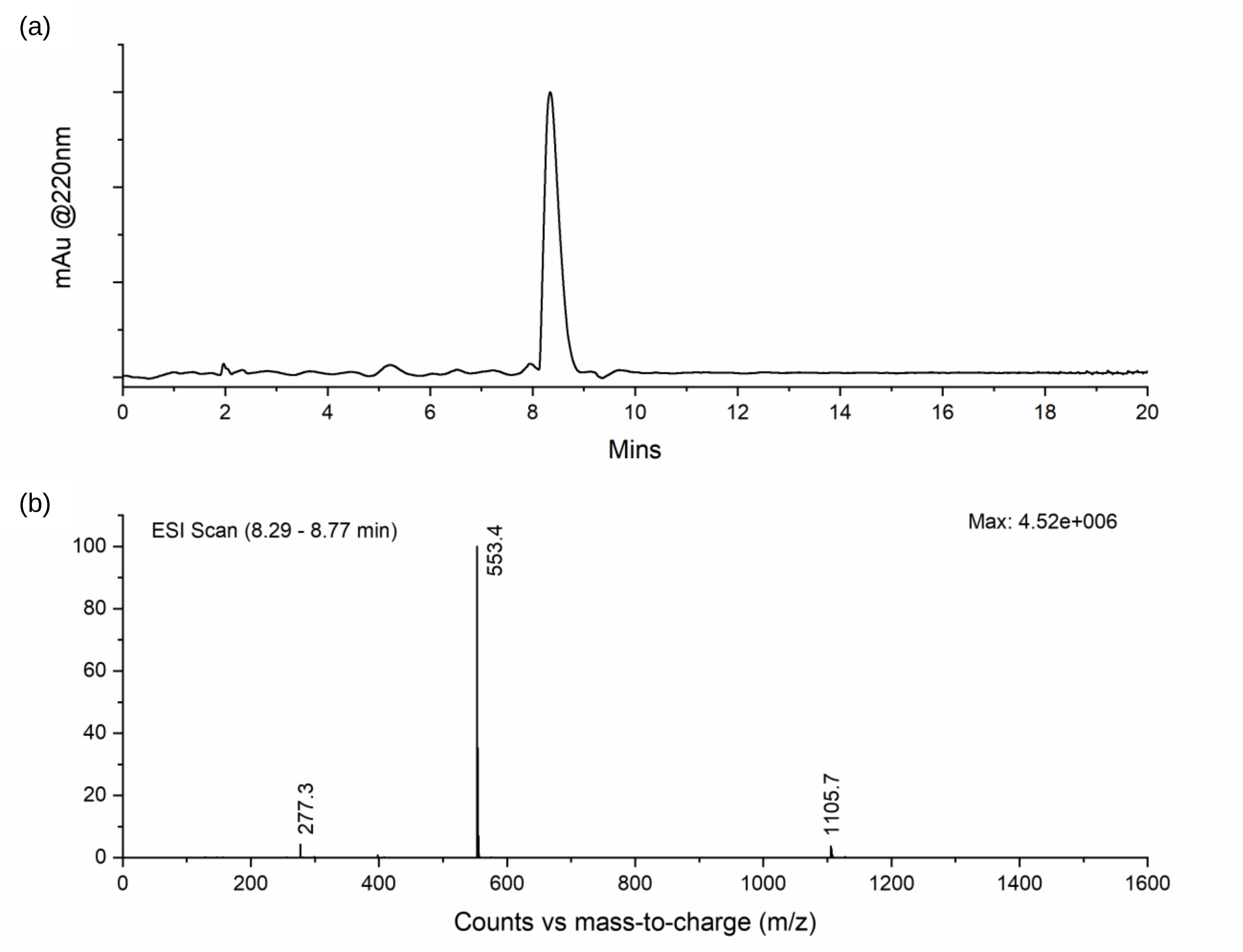

Figure S2. Characterization of IVZK by using LC-MS. (a) Liquid chromatogram of IVFK by the absorbance at 220 nm. (b) Mass spectrum of IVFK. MS: (m/z) calculated 552.8, $[M+2H]^{2+}$ found 277.3, $[M+H]^{+}$ found 553.4, and $[2M+H]^{+}$ found 1105.7.

^{1}H-NMR assignment for IVFK and IVZK in 90% H_2O - 10%D_2O mixture

^{1}H NMR IVFK (600 MHz, H_2O+D_2O) δ 8.46 (d, J = 7.1 Hz, 1H), 8.26 (d, J = 7.7 Hz, 1H), 8.13 (d, J = 2.6 Hz, 1H), 8.12 (d, J = 3.6 Hz, 1H), 7.32 (m, 5H), 6.99 (s, 1H), 6.86 (s, 1H), 4.21 (m, 1H), 4.09 (t, J = 14.6 Hz, 1H), 4.07 (t, J = 8.2 Hz, 1H), 3.06 (m, 3H), 2.96 (t, J = 8.1 Hz, 3H), 2.02 (s, 3H), 1.96 (m, 1H), 1.77 (m, 2H), 1.64 (m, 3H), 1.46 (m, 1H), 1.36 (m, 2H), 1.16 (m, 1H), 0.89 (d, J = 6.9 Hz, 3H), 0.85 (m, 6H), and 0.78 (d, J = 7.0 Hz, 3H)

^{1}H NMR IVZK (600 MHz, H_2O+D_2O) δ 8.39 (d, J = 7.2 Hz, 1H), 8.28 (d, J = 7.5 Hz, 1H), 8.22(d, J = 8.6 Hz, 1H), 8.13 (d, J = 7.5 Hz, 1H), 7.55 (s, 1H), 7.1 (s, 1H), 4.4 (m, 1H), 4.27 (m, 1H), 4.09 (m, 2H), 2.99 (t, J = 8.3 Hz, 3H), 2.02 (s, 3H), 2.01 (m, 1H), 1.8 (m, 3H), 1.64 (m, 9H), 1.45 (m, 3H), 1.32 (m, 1H), 1.18 (m, 4H), and 0.9 (m, 14H)

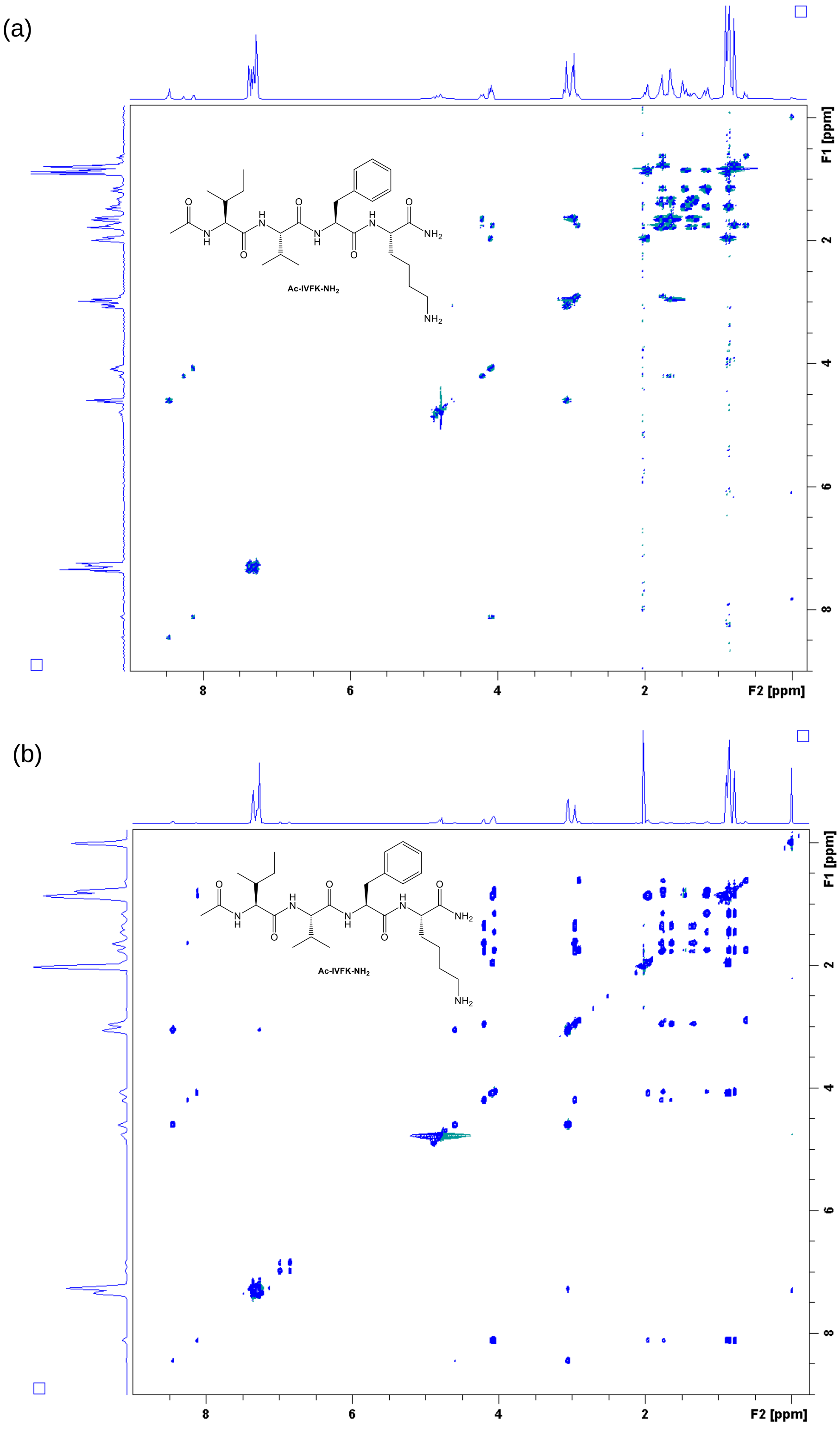

Figure S3. ^{1}H-^{1}H Homonuclear through bond correlations of IVFK. (a) COSY and (b) TOCSY

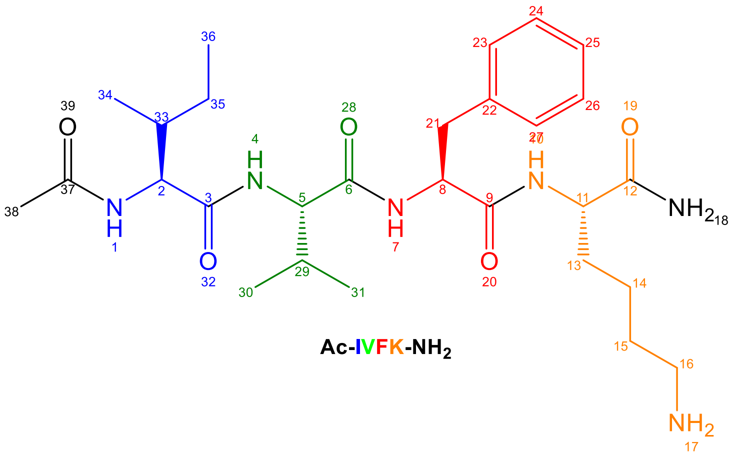

Table S1: 1H-NMR chemical shifts of IVFK

No	δ (ppm 1H)	Splitting	Amino Acid	Atom	Assignment
7	8.46	d, *J* = 7.1 Hz, 1H	F	NH	F_{amide}
10	8.26	d, *J* = 7.7 Hz, 1H	K	NH	K_{amide}
4	8.13	d, *J* = 2.6 Hz, 1H	V	NH	V_{amide}
1	8.12	d, *J* = 3.6 Hz, 1H	I	NH	I_{amide}
22-27	7.32	m, 5H	F	Aromatic	$F_{aromatic}$
18	6.99	s, 1H	C-terminal	NH	Amide Capping
18	6.86	s, 1H	C-terminal	NH	Amide Capping
8	4.62	NI	F	CH	F_{α}
11	4.21	m, 1H	K	CH	K_{α}
5	4.09	t, *J* = 14.6 Hz, 1H	V	CH	V_{α}
2	4.07	t, *J* = 8.2 Hz, 1H	I	CH	I_{α}
21	3.06	m, 3H	F	CH_2	F_{β}
16	2.96	t, *J* = 8.1 Hz, 3H	K	CH_2	K_{ε}
38	2.02	s, 3H	N-terminal	Acetyl	Acetyl capping
29	1.96	m, 1H	V	CH	V_{β}
13	1.77	m, 2H	K	CH	$K_{\beta 1}$
33			I	CH	I_{β}
13	1.64	m, 3H	K	CH_1	$K_{\beta 2}$
15			K	CH2	K_{δ}
35	1.46	m, 1H	I	CH	$I_{\gamma 1}$
14	1.36	m, 2H	K	CH_2	K_{γ}
35	1.16	m, 1H	I	CH	$I_{\gamma 2}$
30	0.89	d, *J* = 6.9 Hz, 3H	V	CH_3	$V_{\gamma 1}$
36	0.85	m, 6H	I	CH_3	I_{δ}
31			V	CH_3	$V_{\gamma 2}$
34	0.78	d, *J* = 7.0 Hz, 3H	I	CH_3	$I_{\gamma 3}$

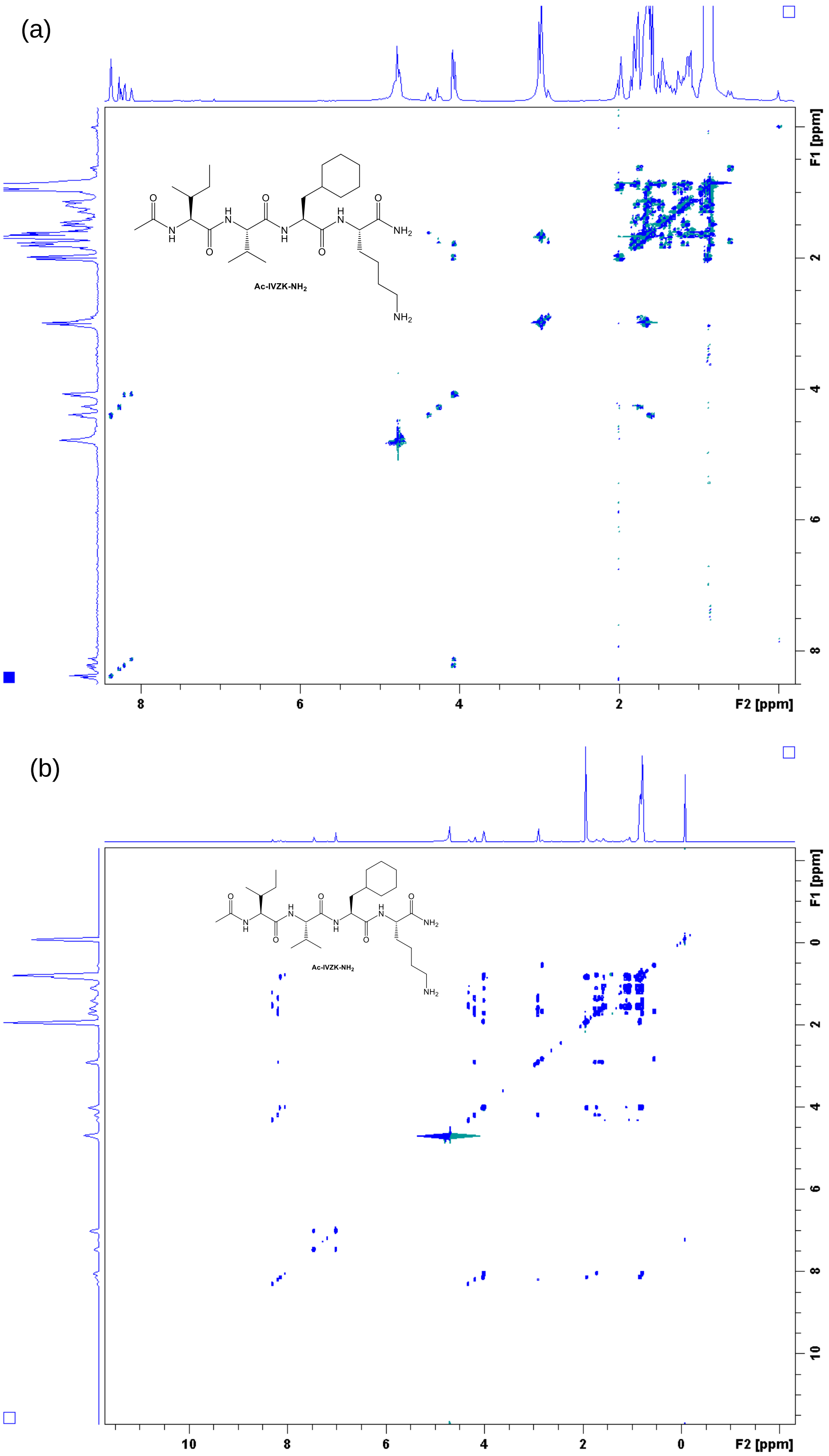

Figure S4. ^{1}H-^{1}H Homonuclear through bond correlations of IVZK. (a) COSY and (b) TOCSY

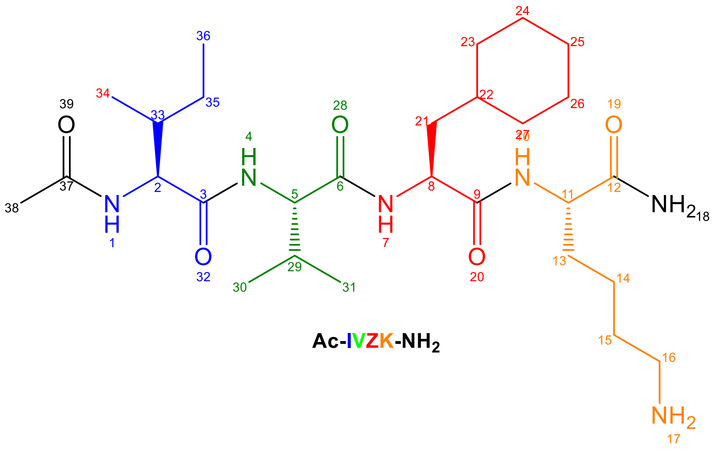

Table S2: 1H-NMR chemical shifts of IVZK

No	δ (ppm 1H)	Splitting	Amino Acid	Atom	Assignment
7	8.39	d, *J* = 7.2 Hz, 1H	Z	NH	Z_{amide}
10	8.28	d, *J* = 7.5 Hz, 1H	K	NH	K_{amide}
4	8.22	d, *J* = 8.6 Hz, 1H	V	NH	V_{amide}
1	8.13	d, *J* = 7.5 Hz, 1H	I	NH	I_{amide}
5	7.55	s, 1H	C-terminal	NH	Amide Capping
6	7.10	s, 1H	C-terminal	NH	Amide Capping
8	4.40	m, 1H	Z	CH	Z_{α}
11	4.27	m, 1H	K	CH	K_{α}
5	4.09	m, 2H	V	CH	V_{α}
2			I	CH	I_{α}
16	2.99	t, *J* = 8.3 Hz, 3H	K	CH_2	K_{ε}
38	2.02	s, 3H	N-terminal	Acetyl	Acetyl capping
29	2.01	m, 1H	V	CH	V_{β}
13	1.8	m, 3H	K	CH_2	K_{β}
33			I	CH	I_{β}
15	1.64	m, 9H	K	CH_2	K_{δ}
21			Z	CH_2	Z_{β}
Z_{ring}			Z	CH/CH_2	Z_{ring}
35	1.45	m, 3H	I	CH	$I_{\gamma 1}$
14			K	CH_2	K_{γ}
Z_{ring}	1.32	m, 1H	Z	CH	Z_{ring}
35	1.18	m, 4H	I	CH	$I_{\gamma 2}$
Z_{ring}			Z	CH/CH_2	Z_{ring}
Z_{ring}	0.90	m, 14H	Z	CH	Z_{ring}
30			V	CH_3	$V_{\gamma 1}$
31			V	CH_3	$V_{\gamma 2}$
Z_{ring}			Z	CH	Z_{ring}
36			I	CH_3	I_{δ}
34			I	CH_3	$I_{\gamma 3}$

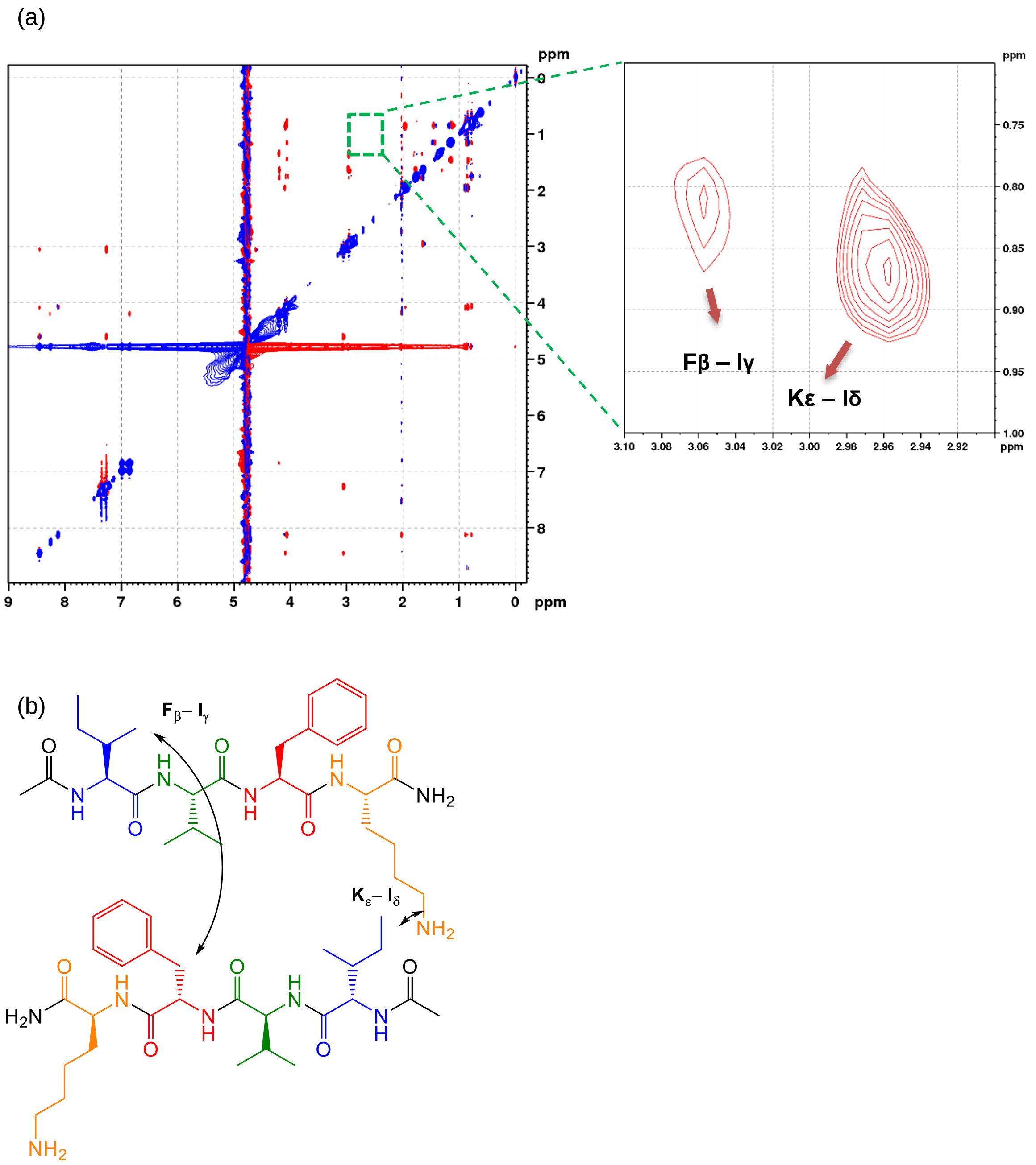

Figure S5. (a) NOESY contour map of IVFK and (b) antiparallel configuration of IVFK based on overlap of NOESY and TOCSY spectra

(a)

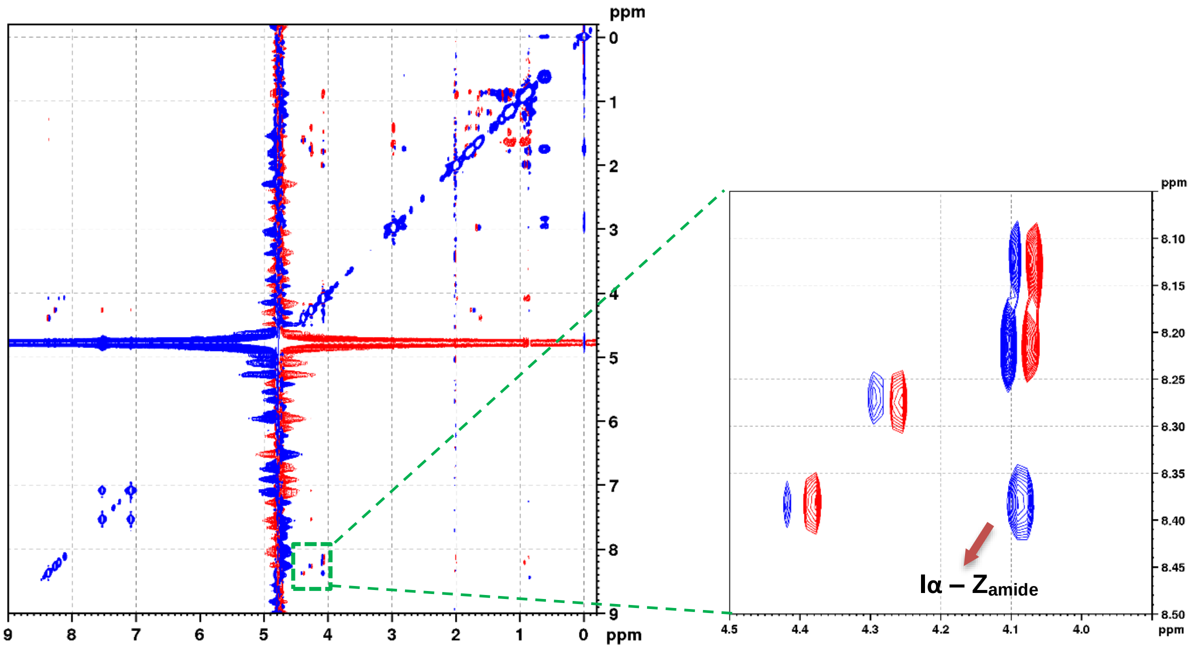

(b)

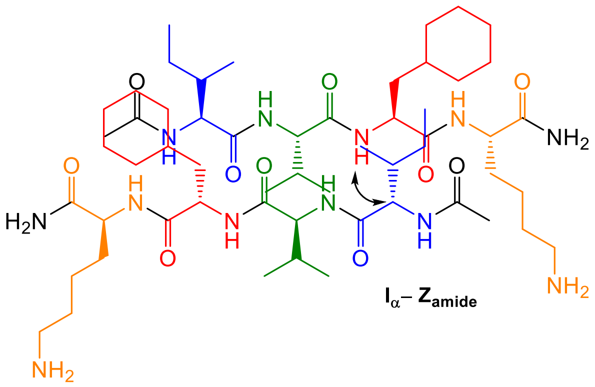

Figure S6. (a) NOESY contour map of IVZK and (b) antiparallel configuration of IVZK based on overlap of NOESY and TOCSY spectra

	1 mg/ml	2 mg/ml	3 mg/ml	4 mg/ml	6 mg/ml	8 mg/ml
IVFK	No gel	< 3 hours	< 20 min	< 5 min	< 1 min	< 1 min
	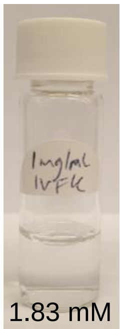	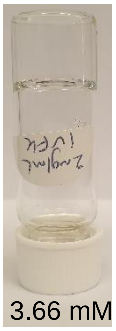	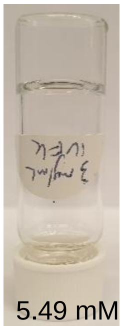	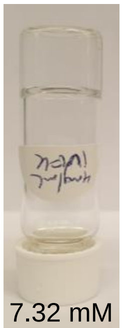	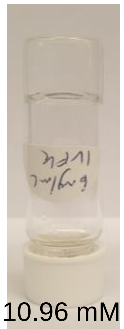	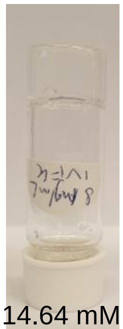
IVZK	No gel	< 15 min	< 5 min	< 5 min	< 1 min	< 1 min
	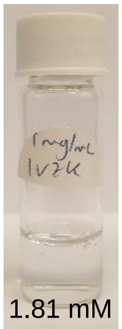	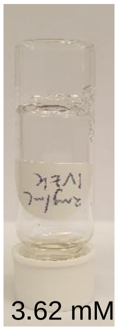	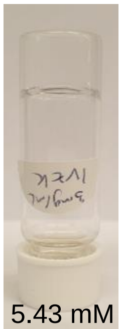	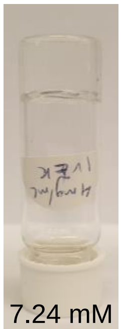	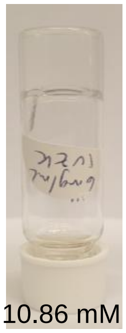	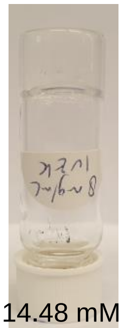

Figure S7. Different concentrations of both peptides in PBS were tested, and the time of gel formation was recorded using inverted tube method.

	Low concentration	High concentration
**IVFK**	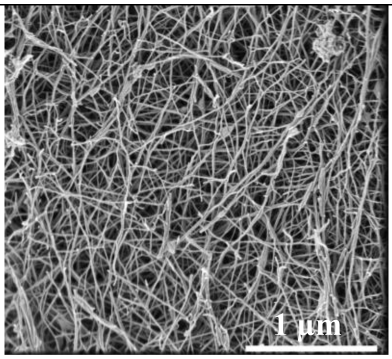 Porosity = 47.43 ± 2.14 %	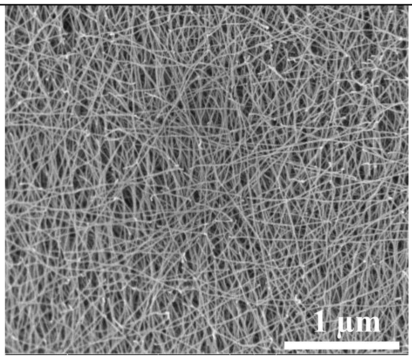 Porosity = 39.04 ± 0.76 %
**IVZK**	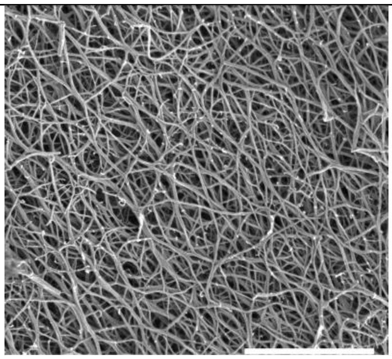 Porosity = 46.91 ± 3.12 %	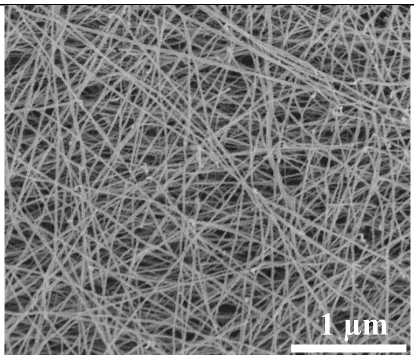 Porosity = 38.42 ± 1.00 %

Figure S8. The porosity of both IVFK and IVZK peptide hydrogels at low concentration (4 mg/mL IVFK and 3 mg/mL IVZK) and high concentration (8 mg/mL for both peptides).

Table S3. Rheological Properties of Peptide Hydrogels at Different Concentrations

Concentration	Storage modulus (kPa)	
	IVFK	IVZK
2 mg/mL	0.54 ± 0.06	10.00 ± 0.12
3 mg/mL	7.03 ± 0.19	17.21 ± 1.38
4 mg/mL	23.40 ± 0.88	29.77± 1.19
6 mg/mL	35.79 ± 0.95	43.03± 2.81
8 mg/mL	51.79 ± 4.73	75.21 ± 4.08

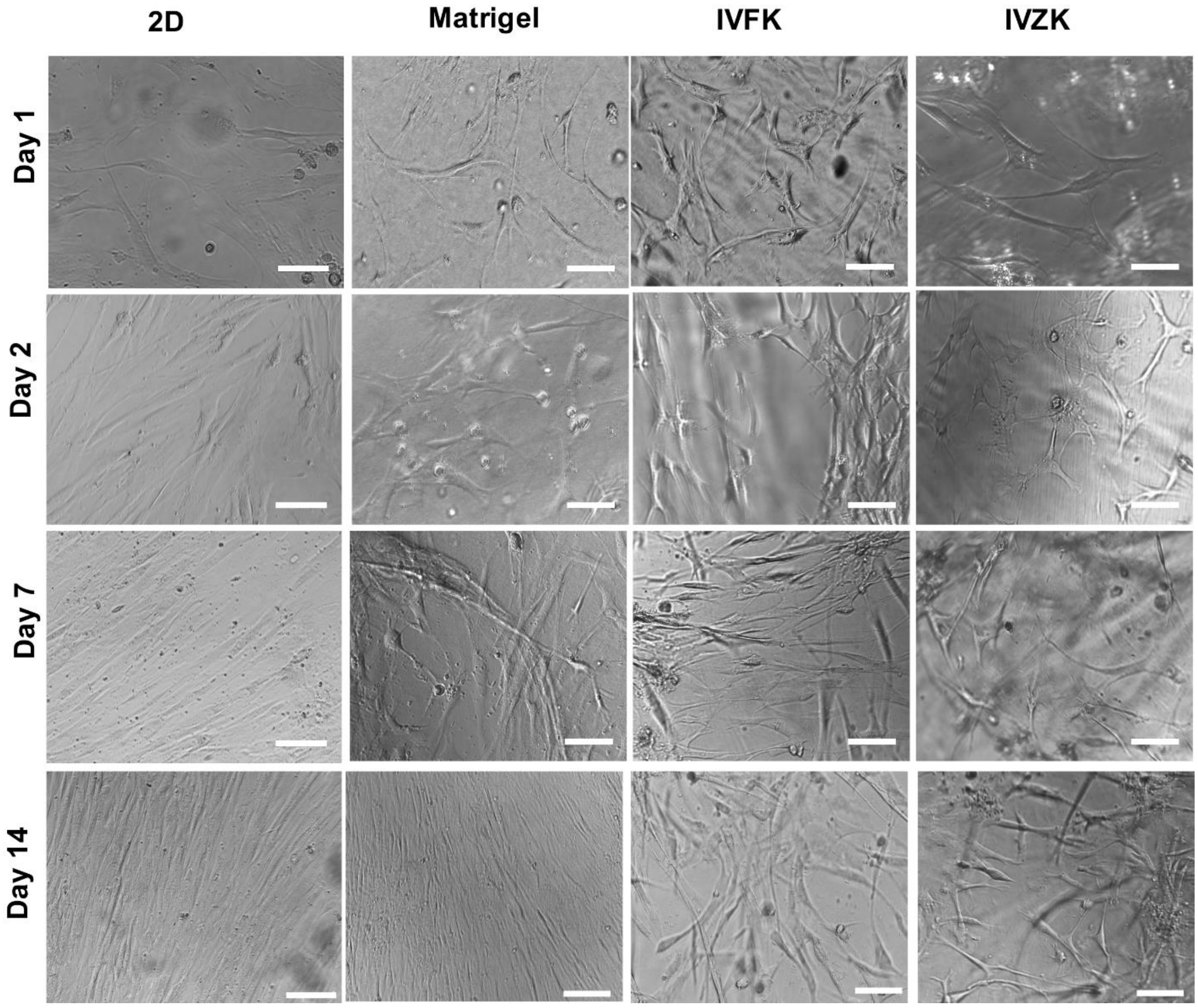

Figure S9. Phase-contrast imaging of BM-MSCs cultured within different scaffolds after 1, 2,7, and 14 days, at which they clearly showed the spindle morphology, network connection between cells, and cell growth increases over time of culture. *Scale bar 100µm*

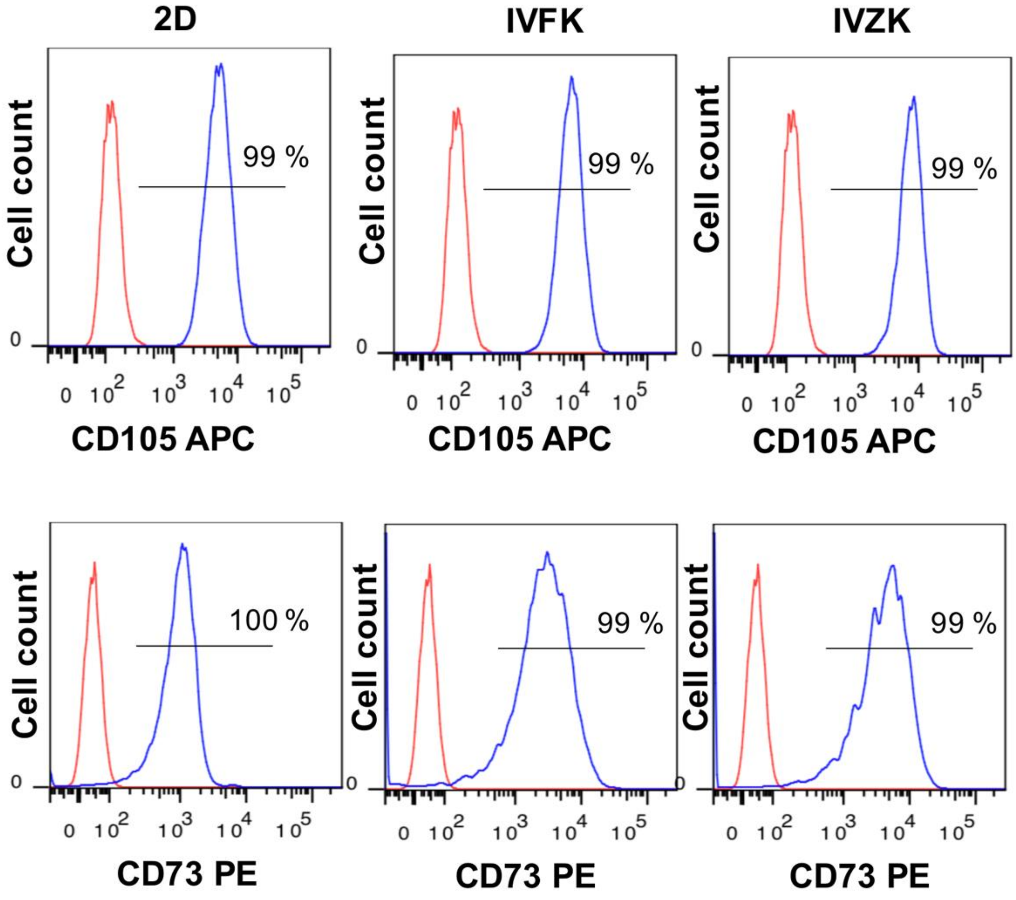

Figure S10. Characterization of BM-MSCs in scaffolds. BM-MSCs were stained against CD73 and CD105. The data shows positive expression of MSC-associated surface antigens CD73, and CD105.

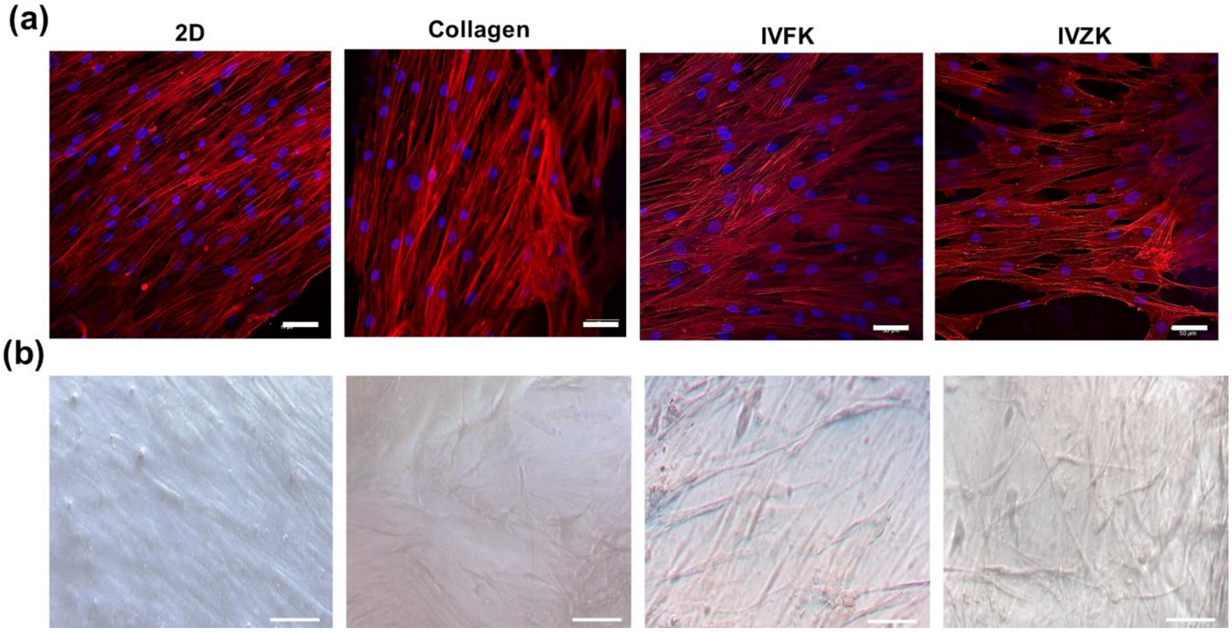

Figure S11. (a) The morphology of hBM-MSCs cultured in normal basal media *Scale bar 50 µm* (b) Alizarin red-S–negative staining of hBM-MSCs cultured in normal basal media. *Scale bar 100µm*

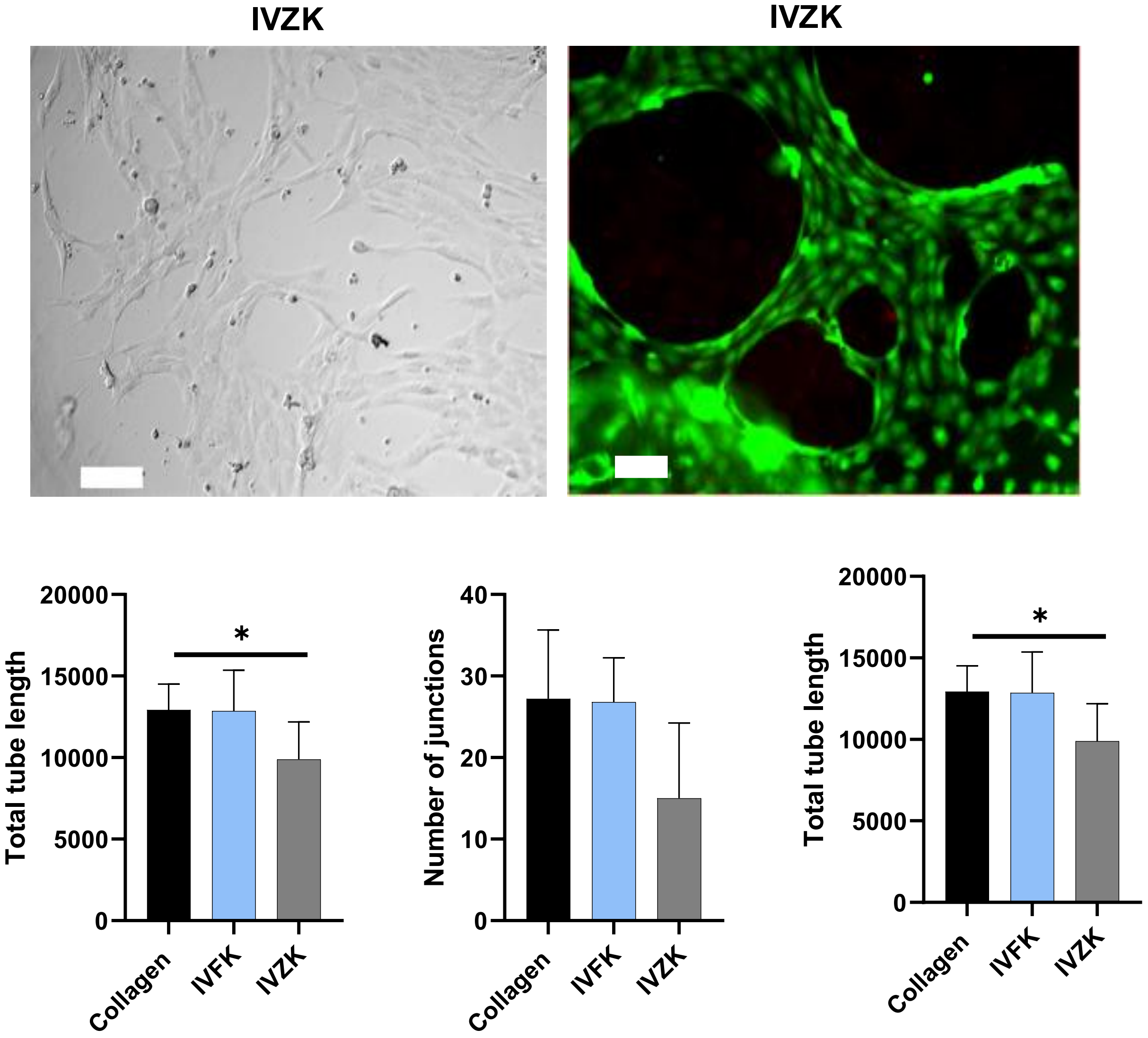

Figure S12. Angiogenesis ability of IVZK scaffold *in vitro.* (a) Calcein-AM (green), Ethidium homodimer (red) staining and bright field image of HUVECs after 24 h, *scale bar 100µm.* (b) Quantification of angiogenesis by measuring vessels junctions, number of nodes and, in total vessels length for 5 different pictures

REFERENCES

1. Hwang, T. L.; Shaka, A. J., Water Suppression That Works. Excitation Sculpting Using Arbitrary Wave-Forms and Pulsed-Field Gradients. *J. Magn. Reson.* **1995,** 112, (2), 275-279.

2. Derome, A. E.; Williamson, M. P., Rapid-Pulsing Artifacts in Double-Quantum-Filtered COSY. *J. Magn. Reson.* **1990,** 88, (1), 177-185.

3. Piotto, M.; Saudek, V.; Sklenář, V., Gradient-Tailored Excitation for Single-Quantum NMR Spectroscopy of Aqueous Solutions. *J. Biomol. NMR* **1992,** 2, (6), 661-665.

4. Sklenar, V.; Piotto, M.; Leppik, R.; Saudek, V., Gradient-Tailored Water Suppression for 1H-15N HSQC Experiments Optimized to Retain Full Sensitivity. *J. Magn. Reson.* **1993,** 102, (2), 241-245.

5. Gilbert, D. F.; Erdmann, G.; Zhang, X.; Fritzsche, A.; Demir, K.; Jaedicke, A.; Muehlenberg, K.; Wanker, E. E.; Boutros, M., A novel multiplex cell viability assay for high-throughput RNAi screening. *PloS One* **2011,** 6, (12), e28338.
